# The Socioeconomic Impact of Transport Costs for Adult Patients Requiring Haemodialysis: A Mixed Methods Study

**DOI:** 10.3390/healthcare12242513

**Published:** 2024-12-11

**Authors:** Sabrina Gröble, Jana Bilger, Chantal Britt, Heidi Kaspar, Sabine Herzig, Kai-Uwe Schmitt

**Affiliations:** 1 Applied Research & Development in Nursing, Department of Health Professions, Bern University of Applied Sciences, 3008 Bern, Switzerland; 2 Institute of Health Economics and Health Policy, Department of Health Professions, Bern University of Applied Sciences, 3008 Bern, Switzerland; jana.bilger@bfh.ch; 3 Competence Centre Participatory Health Care, Department of Health Professions, Bern University of Applied Sciences, 3008 Bern, Switzerland; chantal.britt@bfh.ch (C.B.); heidi.kaspar@bfh.ch (H.K.); 4 Department of Nephrology and Hypertension, Inselspital, Bern University Hospital, 3010 Bern, Switzerland; sabine.herzig@insel.ch; 5 Academic-Practice-Partnership of Bern University of Applied Sciences and Insel Gruppe, Bern University Hospital, 3008 Bern, Switzerland; kai-uwe.schmitt@bfh.ch

**Keywords:** haemodialysis, dialysis, transport, costs, financial burden, social impact

## Abstract

Background/Objectives: Patients requiring haemodialysis often perceive the cost of their travels to the dialysis centres as a significant burden. The study aimed to collect a first Swiss national data set on transport costs and assess their impact on patients and their relatives. Methods: In addition to interviews with patients, a quantitative survey was developed and distributed online using a voluntary sampling strategy. Data were analysed by means of descriptive statistics. A Sounding Board of patients, relatives, and healthcare professionals accompanied all steps of the project. Results: A total of 6 dialysis patients were interviewed; 437 respondents of the national survey met the inclusion criteria. Patients travel a median distance of 7.5 km to their dialysis centres. For 78% of them, the journey takes up to 30 min, and nearly half use their private car as their main mode of transport. The median annual transport costs are CHF 2925 (mean = CHF 5041). Approximately half of the patients perceive transport costs as a burden or limitation, yet only about 30% receive financial support, for which there is no standard process. Patients requiring financial support reported difficulties navigating the system. Conclusions: This study highlighted that many dialysis patients are burdened by transport costs and receive limited financial support and counselling services. Implementation of a standardised process for financial support should be explored.

## 1. Background and Objectives

Dialysis is a life-saving treatment for patients with chronic kidney disease. Chronic kidney disease is one of the leading causes of mortality affecting more than 10% of the general population worldwide [1]. In Switzerland, around 4800 persons with end-stage kidney disease receive dialysis, with a mean age of 70.1 years and men being affected more frequently (67.8%) than women (32.2%) [2]. Many dialysis patients are multimorbid, as chronic kidney failure is often the result of prevalent systemic diseases such as arterial hypertension, obesity, or diabetes mellitus [3,4].

There are currently two modalities of dialysis treatments available: haemodialysis and peritoneal dialysis. Haemodialysis can be performed in a centre, in a specialised practice, or at home, while peritoneal dialysis is usually administered at home [5]. The choice between these treatments depends on the patient’s medical needs, availability of and access to modalities, personal abilities, and family support, and is ideally made together with the patient, their family, and healthcare professionals [5,6,7]. According to data from 59 countries, in-centre haemodialysis is the most widely used treatment modality worldwide [8]. In Switzerland, approximately 90% of the patients receive haemodialysis in a dialysis centre or specialised practice [3].

Despite its life-saving role, dialysis can impose a significant social and financial burden on patients and their families. Haemodialysis typically requires three four-hour treatments per week [2,5], which is both time-consuming and physically demanding. After dialysis, patients often report symptoms such as muscle cramps, extreme fatigue, cognitive dysfunction, and hypotension [9], which can further limit their functions. For dialysis patients of working age, the time-consuming and physically demanding nature of the treatment, combined with its impact on their health, often means that they are frequently unable to work full-time, which reduces their household income [9,10,11]. If patients lose their jobs, are forced to relocate for their treatment, or are unable to travel to or participate in social events due to their illness and therapy, dialysis may lead to social isolation. Disability can furthermore lead to patients becoming increasingly dependent on others, which is a potential burden for patients. Relatives are involved in the care of dialysis patients in a variety of ways: they manage medical treatments, help with dietary requirements, help arrange appointments for medical visits or dialysis, or take care of patient transport [9,11]. Such long-term dialysis-related care tasks can lead to caregiver burden in dialysis patients’ relatives, which may be associated with increased stress levels, depression, or anxiety [12,13].

As the subsequent description illustrates, transport to dialysis treatments and the associated costs can be a challenge for patients and their families. Patients rely on various modes of transport to travel to dialysis, including private cars, public transport, transport services (e.g., taxi), volunteer or subsidised transport services (e.g., Swiss Red Cross), or ambulance services. A Canadian study suggests that the choice of the mode of transport depends on factors such as the patient’s financial situation and health status, including the occurrence of post-dialysis symptoms mentioned above. Patients also described the challenge of travelling to and from treatment centres several times a week. They reported problems with public transport, increased feelings of anxiety and frustration, and difficult weather conditions during travel [9].

Research from different European countries has shown that transport costs related to haemodialysis can be substantial. For example, a Dutch study reported average annual transport costs of EUR 5455 (standard deviation (SD) = 5499) for patients using taxi services [14]. In France, transport costs for different modes of transport ranged from EUR 900 (private car) to EUR 10,000 (ambulance) per year [15]. In the UK, a cost model estimated transport costs to be around EUR 4499 (£3773) per year [16]. The amount that dialysis patients must pay out of pocket varies between countries. However, the financial burden of transport costs can have a negative impact on patients’ health and social participation. For example, a change in financial status was found to be associated with worsening symptoms such as psychosocial stress, fatigue or pain, as well as increased levels of depression and sexual dysfunction [17].

To date, no data on haemodialysis transport costs have been collected in Switzerland. Although the basic health insurance is compulsory for Swiss residents, it can be assumed that the transport costs are high for patients, as the basic insurance covers only half of the transport costs, up to a maximum of CHF 500 per year if the patient provides a medical certificate. However, if a private car is used, the costs are usually not covered [18]. Depending on the individual situation, costs not covered by the basic insurance may be reimbursed by supplementary insurance (if available), disability benefits, accident insurance, social benefits, or supports from private foundations [18,19].

In summary, current studies indicate that transport costs can be a significant burden for dialysis patients, negatively affecting their and their relatives’ social participation, health, and well-being. The aims of the present study were therefore (1) to collect the first national data set on transport costs in Switzerland and (2) to better understand the effects of transport costs on patients and their relatives in a context where the frequent and potentially long journeys required to access the life-saving therapy are not covered by compulsory health insurance.

## 2. Methods

A sequential mixed methods design was used to answer the research question: In a first step, exploratory semi-structured interviews were conducted with dialysis patients (see Section 2.2). In a second step, the results of the interviews were used to develop a questionnaire for a national online survey (see Section 2.3). Both, the qualitative and quantitative phases, benefited from the expertise and practical experiences of patients, their families, and healthcare professionals (see Section 2.1). In a third step, the results of the qualitative and quantitative studies were synthesised.

### 2.1. Sounding Board

Research gains in quality, appropriateness, and relevance when involving target groups and important stakeholders were targeted [20]. Therefore, a Sounding Board was formed for this project, consisting of four dialysis patients: one wife of a patient and three healthcare professionals. During regular meetings, the project’s status was discussed. Thus, the group ensured that the views and experiences of patients, relatives, and healthcare professionals were considered in all phases of the project. The Sounding Board provided feedback on the survey instruments, the procedure for conducting the national online survey, and participant recruitment. Additionally, the Sounding Board supported the project team in the interpretation of the qualitative and quantitative data by identifying potential explanations for the observed study results.

### 2.2. Qualitative Part: Interviews

The purpose of the qualitative interviews was to explore known findings from the literature in a Swiss context and gain more insight from the patients’ perspective. The interviews guided the research team in the formulation of the online survey’s questions, the definition of the study’s primary objectives, the prioritisation of the topics, and provided feedback on findings from the literature review.

#### 2.2.1. Recruitment

In line with the purpose of the interviews, a small sample size of five patients was sought. The aim was to obtain a sample that was as diverse as possible in terms of age, mode of transport, gender, and socioeconomic status. In addition, patients from urban and rural areas should be included. Participants for the interviews were recruited through healthcare professionals working in dialysis centres and their networks. Patients were included if they were dependent on haemodialysis, were over 18 years of age, had no cognitive impairment, and could understand and speak German, Italian, French, Rhaeto-Romanic, or English. Whenever possible, the relative who accompanied the recruited patients during transport was also interviewed. All subjects were informed about the study, both verbally and in writing, and gave their written consent.

#### 2.2.2. Data Collection

A semi-structured interview guide was developed based on the literature and included questions about the transport and its impact on the patient’s financial situation, social life, physical and mental health, and the person who accompanied the patient during the transport. The guide was discussed, complemented, and validated by the Sounding Board. The interviews, which lasted between 30 and 45 min, were conducted in May 2023 by the first author either in dialysis wards or at the patients’ homes, depending on the participants’ preference. They were documented in handwritten notes.

#### 2.2.3. Data Analysis

As the interviews were primarily conducted to prepare for the subsequent online questionnaire, the analysis presented here was not the focus of the research. Consequently, the analysis followed a less thorough approach in that respect, and it was deemed appropriate to adopt a pragmatic analysis approach, incorporating elements of content analysis as described by Kuckartz [21]. The first step involved the first author reading the handwritten notes and applying deductive coding based on predefined categories derived from the existing literature, which were also utilised in the semi-structured interview guide. Next, the notes were re-examined to inductively refine the coding by complementing the initial categories and developing subcategories (see Table A1, Appendix A). The content of each category was then summarised in written form. Subsequently, these findings were discussed with the second author and processed for presentation and discussion with the Sounding Board. The collective findings and conclusions from the final debates with the Sounding Board were summarised in a short report, which formed the basis for the formulation of the questions or response options for the national online survey. Table A1, Appendix A shows which interview topics influenced the online survey and which question(s) were formulated with their help.

### 2.3. Quantitative Part: Online Survey

A national survey was developed and deployed to collect data related to transport costs and their impact.

#### 2.3.1. Recruitment

The target population comprised patients aged 18 years and older who required haemodialysis at least once a week. A voluntary sampling strategy was implemented. The Swiss Kidney Foundation informed all 109 dialysis centres in Switzerland and 123 members of the Swiss Society of Nephrology about the study by email and asked them to forward the study information to dialysis patients. A reminder was sent to dialysis centres and society members three weeks after the initial information on the study. Two associations (Swiss Kidney Patients Association, Nephrology Care Interest Group) and Bern University of Applied Sciences disseminated information about the study through various channels, such as blog posts, website articles, and social media. Consent to participate was obtained digitally at the beginning of the online questionnaire.

#### 2.3.2. Data Collection

The primary focus was transport costs incurred for the travels to and from the dialysis facilities. Secondary outcomes included details on transport modalities, transport needs, and potential consequences of costs for patients. As to our knowledge, no suitable questionnaire existed, the study team developed a questionnaire based on insights from the qualitative interviews and the relevant literature, including similar questionnaires identified in a pragmatic literature search [21,22]. The anonymised questionnaire consisted of 28 to 44 questions, varying on the number of follow-up questions triggered by the respondents’ answers. To prevent order bias, answer options were randomised where applicable. Survey items included questions on sociodemographic characteristics, dialysis treatment, transport, costs, social factors, and counselling. To learn about the transport modes, we assessed the number of different transport modes someone used per journey and the main mode of transport. Transport costs were calculated for the main mode of transport and are reported in Swiss Francs (CHF).

For patients using public transport, taxis, patient transport, or voluntary transport as the main mode of transport, costs were derived from reported (ticket) costs for the previous one-way trip and the number of dialysis sessions per year to avoid recall bias. For those using public transport, annual costs were capped at CHF 3860, which corresponds to the price of an annual travel pass valid on the entire Swiss public transport network. For those using private cars or motorcycles as their main mode of transport, costs were calculated based on reported distances to the dialysis centres in kilometres, the costs per vehicle per kilometre as provided by the Touring Club Switzerland [23] and the Federal Statistical Office [24], respectively, and the number of dialysis sessions per year. The calculation basis for all modes of transport is presented in Table A2, Appendix B.

The questionnaire was developed in German, discussed and finalised with the Sounding Board, translated, and made available in the seven most frequently spoken languages in Switzerland, namely German, French, Italian, English (available in Appendix C), Portuguese, Albanian, and Spanish [25]. The survey was conducted online from 3 July to 14 August 2023 using LimeSurvey software (LimeSurvey GmbH, Hamburg, Germany, version 2.56.1).

#### 2.3.3. Data Analysis

The entire analysis was conducted with the statistical software R (version 4.3.1, R Foundation, Vienna University of Economics and Business, Vienna, Austria). The survey results were analysed using descriptive statistics, and non-parametric tests were used to assess differences between subgroups (see corresponding tables in the Appendix A). Responses were included in the analysis, regardless of whether the survey was completed or not. However, missing responses were analysed at both item and case levels, and cases with more than 10% missing responses were excluded from the analysis. No imputation techniques were applied, which led to varying numbers of respondents for different questions. To prevent implausible responses from biasing the results, free text responses relevant to cost calculations (e.g., ticket cost in Swiss Francs) were checked for plausibility. Responses deemed implausible based on criteria defined by the project team were excluded from the analysis of transport costs. The Sounding Board supported the project team in interpreting the observed survey results.

#### 2.3.4. Representativity

The “Swiss Renal Registry and Quality Assessment Program” (SRRQAP) of the Swiss Society of Nephrology is a registry capturing all chronic dialysis patients in Switzerland [2]. To learn about the representativeness of the survey results, we employed statistical comparison tests to determine if our sample significantly differed from the population on observable attributes. We compared aggregated data (i.e., proportions, means, SD) on age, years requiring dialysis, number of dialyses per week, gender, nationality (Swiss, EU, other), and Swiss language region (German, French, Italian) from the latest registry data of 2021 with the survey data (see Table A3, Appendix D). No adjustments, such as weighting, were applied to address potential representativeness issues.

#### 2.3.5. Ethical Consideration

This research was conducted in line with the Helsinki Declaration (2013 rev.). Local legislation did not require formal ethical approval. All data were treated confidentially and was only accessible to the study team.

## 3. Results

### 3.1. Study Population

Six dialysis patients from different urban and rural regions of the country were recruited for the interviews (see Table A4, Appendix E). Five interviews were conducted in three different hospitals on their dialysis ward, and one interview took place at a patient’s home. The interviews were conducted in German (n = 4), in Italian (n = 1), and in Rhaeto-Romanic (n = 1). The participants, four men and two women, were between 50 and 83 years old (mean = 75.5) and had been undergoing haemodialysis at a centre three times a week between 0.5 and 10 years (mean = 3.3). Five of the six patients were retired; the sixth, who was of working age, was looking for a new job at the time of the interviews, as her previous job was not compatible with three dialysis treatments a week. Three of the patients were accompanied to and from the dialysis centre by relatives or friends. Two accompanying persons, both spouses (one male, one female), also participated in the interviews. The accompanying person of the remaining four patients could not be interviewed because they could not be present at the time of the interview or did not wish to participate.

**Figure 1:**
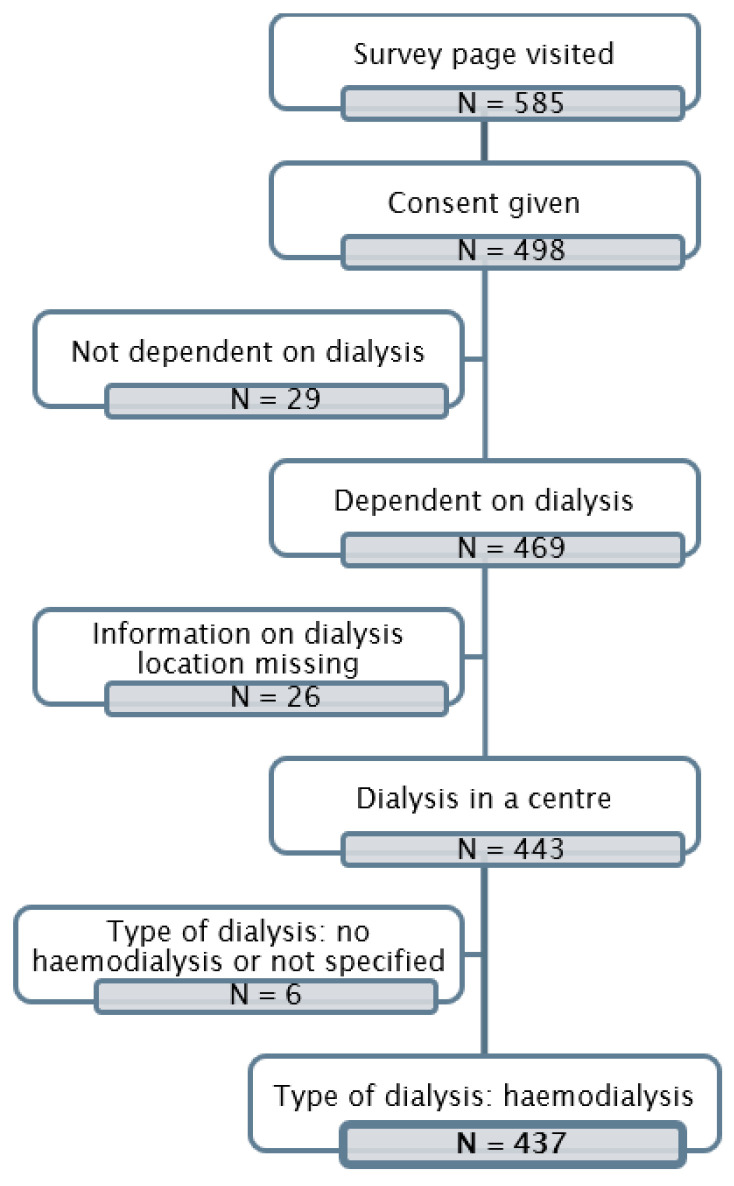
Flow chart for the inclusion and exclusion of the survey participants.

A total of 498 persons participated in the national online survey. Among those who responded, 66.5% answered the questionnaire in German, 23.1% in Italian, and 10.4% in the other available languages. A total of 41.9% of respondents stated that they had someone assisting them in completing the online survey. A total of 469 of the 498 respondents confirmed that they were dialysis patients, i.e., more than 10% of dialysis patients registered in Switzerland participated in the online survey. A total of 437 respondents were undergoing dialysis in a centre and thus met the inclusion criteria (Figure 1). Sixty-six observations (15.1%) were excluded from the subsequent analysis due to the proportion of missing information. Finally, survey results from a total of 371 dialysis patients were analysed. The results presented in the following refer to this population.

The mean age of the survey respondents was 70.1 years (SD = 14.2); 250 were men (67.8%) and 119 were women (32.2%). Most of the respondents (n = 271, 73.6%) had been on dialysis for 0 to 5 years, 17.4% (n = 64) for more than 5 to 10 years, and 9.0% (n = 33) for more than 10 years. The mean weekly session frequency was 2.9 (SD = 0.3). The comparison with the SRRQAP dialysis register [2] showed that the study population reflects the Swiss dialysis population well in terms of age and years of dialysis (see Table A3, Appendix D). However, persons without Swiss or European passports, patients from French-speaking Switzerland, and women were underrepresented in the survey, while patients from Italian-speaking Switzerland were overrepresented. Most participants were retired (n = 324, 87.8%) and 6.7% (n = 25) lived in a care institution.

Most respondents (n = 271, 73.8%) stated that they had chosen their dialysis centre based on recommendations from healthcare professionals after the need for dialysis had been established. The majority (n = 295, 79.7%) selected the centre nearest to their home. Fifty-one patients (13.8%) knew there was a closer centre but did not select it mainly because of “personal preference” or “medical reasons”. The qualitative interviews revealed a more detailed picture: one person, for instance, selected one of two available centres as it did not require him to change trains during his journey, as the stairs at the train station posed a challenge for him. Another interviewee explained that although there was a dialysis centre much closer in a neighbouring country, it was not an option for her, because of concerns about an inferior quality of medical care provided. As a result, it took her up to two hours to travel one way to the nearest dialysis centre in Switzerland, which is three times longer than the journey to the centre in the neighbouring country.

### 3.2. Travel and Modes of Transport

**Figure 2:**
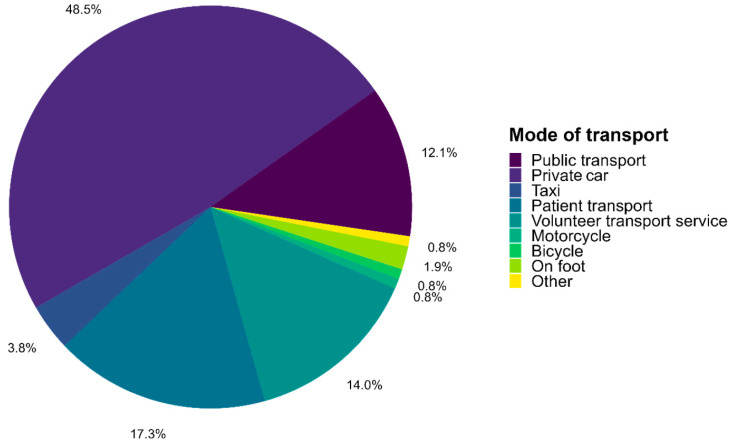
Main modes of transport used by dialysis patients in Switzerland. N = 348, N total = 371.

Survey respondents travelled a median distance of 7.5 km (IQR 3.5–17.5 km; mean = 11.9 km, SD = 10.6 km) one way to or from their dialysis centre, with the one-way journey taking less than 30 min for most participants (n = 286, 77.7%). For 20.1% (n = 74) of cases, the journey took between 0.5 and 1 h, and for a few, it took between 1 and 1.5 h (n = 6, 1.6%) or more than 2 h (n = 2, 0.5%). Most of the respondents travelled with only one mode of transport (n = 334, 90.0%) and used the same mode of transport for the outward and return journey (n = 360, 97.0%). Figure 2 shows that the private car was the most frequently used main mode of transport (n = 180, 48.5%), followed by patient transport (n = 64, 17.3%). A volunteer transport service was used by 14.0% (n = 52) of dialysis patients and public transport by 12.1% (n = 45). Almost half (n = 176, 48.1%) of the respondents did not travel alone to dialysis centres. Of these, 78.4% (n = 138) (i.e., 37.7% of the total survey sample) had to be accompanied, either by someone from their private social network like friends or relatives (n = 49, 35.5%) or by a driver or transport service employee (n = 89, 64.5%). Patients relied on private accompaniment due to “non-dialysis-related health limitations” (n = 36, 73.5%), “post-dialysis discomfort” (n = 17, 34.7%), “pre-dialysis kidney discomfort” (n = 10, 20.4%), or “other reasons” (n = 9, 18.4%) (multiple choices possible).

Patients who travelled by private car reported that this was the “easiest/most convenient solution” for them (n = 109, 61.9%). The “shortest travel time” (n = 67, 38.1%) was also a significant factor in decision making (multiple choices possible). Among respondents using patient transport, the majority indicated that this mode of transport was the “easiest/most convenient solution” (n = 31, 51.7%). In addition, “non-dialysis-related physical limitations” were a frequent reason (n = 29, 48.3%) for using transport services. Volunteer transport services were often used because of “non-dialysis-related physical limitations” (n = 32, 65.3%) and/or “dialysis-related symptoms” (n = 17, 34.7%). When using public transport, the most reported reasons were “easiest, most convenient solution” (n = 20, 46.5%), “no driving license” (n = 11, 25.6%), and “travel costs” (n = 11, 25.6%). Most respondents (n = 273, 74.9%) were satisfied or fairly satisfied with their current transport situation. A total of 53.0% (n = 195) of the respondents stated that they had received advice on transport options after dialysis became compulsory. A total of 22.3% (n = 82) had not received advice but wished they had. The remaining 24.7% (n = 91) had not received advice but were also not interested in it.

The interview partners used various modes of transport, including private cars, volunteer transport services, taxis, and public transport. The reasons for choosing the means of transport did not differ from the online survey, and the interviewees expressed satisfaction with their transport situation. Nevertheless, some challenges related to transport were also mentioned, as the following example illustrates:

*‘If my accompanying person is spontaneously unavailable, it is almost impossible to organise volunteer transport as it must be booked in advance. Therefore, I must rely on a taxi. However, as a taxi does not have a care mandate, I must hope that the driver is willing to accompany me on foot from the car park to the centre.’ (Interview Patient 2)*

Interviewees who currently travel independently also reported that they dreaded the moment when they could no longer do so because they did not know how they would be able to afford the cost of a (volunteer) transport service.

### 3.3. Transport Costs

**Figure 3:**
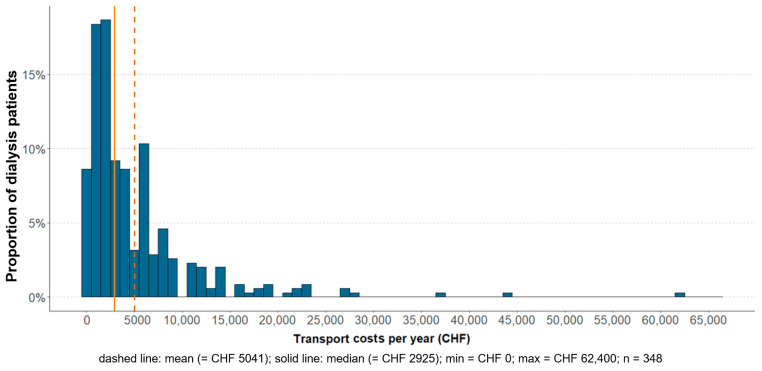
Transport cost per year and dialysis patient in Swiss Francs (CHF). The mathematical formulae for the calculation of the transport costs for the different modes of transport can be found in Table A2, Appendix B.

Survey results showed that the median annual travel expenses were CHF 2925 (IQR 1170–6006) before the deduction of financial support. Figure 3 illustrates the high skewness of the costs, with a mean expense of CHF 5041 (SD = 6554) per patient (n = 348).

Annual costs varied considerably between the different modes of transport. Respondents traveling by taxi had the highest median expenses (CHF 9828, IQR 6552–14,040; mean = CHF 10,460; SD = 7336), followed by patients traveling by volunteer transport (median = CHF 7488, IQR 874–12,480; mean = CHF 9381, SD = 6685) and patient transport (median = 6396, IQR 2496–12,480; mean = CHF 10,200, SD = 11,836) (see Table A5, Appendix F for details).

One of the interviewees explained that he had initially travelled (55 km each way) with a volunteer transport service which cost CHF 140 per dialysis session. Due to the high costs, he had switched to public transport. The idea of needing volunteer transport again if his health deteriorated worried him, also because there is no dialysis centre closer to his home. Other interviewees expressed similar concerns.

### 3.4. Financial Support and Burden

Most of the respondents (n = 252, 70.8%) reported not receiving any financial support for travel costs. Of the 29.2% (n = 104) who reported that they were financially supported for travel costs, only 86 persons provided information on the amount, which resulted in a median of CHF 2000 (IQR 525–5150) per year. To assess the financial situation of dialysis patients, an equivalent income was calculated, which takes into account the weighted income of all persons living in the household (see Table A6, Appendix G). About a third (n = 100, 31.0%) of the respondents who had provided the required information (N = 322) had a monthly equivalent income of ≤ CHF 3000, 39.1% (n = 126) had an equivalent income of CHF 3001–5000, and 24.8% (n = 80) had an equivalent income of CHF 5001–10,000. Only 5.0% (n = 16) had an equivalent income of more than CHF 10,000.

Respondents received financial support for transport costs from their compulsory health insurance (n = 38, 37.6%), followed by supplementary health insurance (n = 34, 33.7%), other sources (n = 23, 22.8%), supplementary benefits (n = 18, 17.8%), disability insurance (n = 7, 6.9%), and foundations (n = 5, 5.0%). None reported receiving financial support from accident insurance or individuals (e.g., friends or relatives). Patients who received financial support had significantly higher median transport costs compared to patients without financial support (see Table A6, Appendix G). Persons with a lower equivalent income more often received financial support compared to those with a higher income, but there is no statistically significant difference in costs between these groups. 45.0% (n = 165) of respondents had received advice from a specialist about financial support for transport after becoming dependent on dialysis. Nonetheless, 36.0% (n = 132) said they would have liked to but had never received such advice. The proportion of those who received financial support was higher in the group who had received advice (n = 74, 46.8%) than in the group who had not (n = 29, 15.0%).

Most respondents (n = 283, 78.2%) agreed or tended to agree with the statement that they would like to receive (more) financial support for transport costs. This wish was expressed slightly more often in the group of those not receiving financial support (n = 198, 80.8%) than in the group of those receiving financial support (n = 72, 70.6%). Approximately half of respondents agreed or tended to agree with the statements that the cost of transport to dialysis caused them to make financial restrictions in their daily lives (n = 159, 44.0%) or that the cost of transport to dialysis caused them to worry (n = 185, 50.9%).

Of the patients interviewed, three (travelling by private car, taxi, volunteer or public transport) did not feel financially burdened and therefore did not apply for support. Two of the others received financial support from their health insurance and one from her disability insurance. However, for these patients, support payments did not cover all costs, and the husband of the patient using the private car (usually not supported) described the following problem:

*‘I take my wife to dialysis, then go home and do the housework because I work later in the afternoon. Then I pick her up. We receive CHF 0.30 per kilometre from the basic insurance for the journey to and from the centre. The journey home in between is not reimbursed. Others, e.g., persons from transport services receive CHF 0.60 per kilometre [note: the Touring Club Switzerland [23] generally calculates the real average cost of CHF 0.75 per kilometre]. We don’t understand why there is such a difference. We have tried to talk to the health insurance company, but they told us that we should be happy to get anything at all. In addition, the parking space at the dialysis centre costs us CHF 180 a year. The high costs are a heavy financial burden for us, and we must cut back financially in our daily lives.’ (Interview 1)*

Other interviewees also reported about restrictions in daily life to be able to afford transport. Two other patients explained that parking at their centre was free, which they perceived as a financial benefit.

### 3.5. Impact on Mental Health and Social Life

Only a minority of respondents believe that transport to dialysis has a (rather) negative impact on their mental health (n = 77, 20.8%). Many reported to not notice any influence (n = 113, 30.5%) or rated it as (rather) positive (n = 180, 48.6%). Most of the interviewees also reported no or rather positive effects of transport on their mental health. Of the interviewees, only one patient said that sometimes everything was “too much” for her. Nevertheless, she said that this referred not specifically to transport but to the dialysis situation in general.

Almost half of the survey respondents (n = 180, 48.6%) reported that having to travel to dialysis centres (rather) positively affected their relationship with relatives, partners, or friends. For 15.2% (n = 56), the impact was (rather) negative, and for 36.2% (n = 134), transport had no impact on social relationships. In the interviews, more detailed aspects of the social impact were reported. The three patients who were accompanied by family members appreciated the travel time as an opportunity to cultivate their relationships. One of the interviewees reported the following:

*‘I’m often tired, so I don’t get as many visitors as I used to, and I don’t go to as many events either. When the transport service is cancelled or my wife can’t drive me to haemodialysis, a friend drives me. We’ve been in touch a lot more since this situation started, and we both really appreciate the time we spend together on the drive.’ (Interview Patient 2)*

The patients also expressed concern that it might be too much for their partners or families and that, as a patient, it is sometimes difficult to depend on others. The interviewed relatives also appreciated the time spent with the patients and the variety it brought to their daily lives. At the same time, they described stress associated with transport, such as the fixed structures of daily life or difficult traffic conditions. However, this information was not always communicated to patients as not to cause concern. The interviews revealed further implications for social life. For instance, one relative had to reorganise his working day to be able to accompany his wife. Other patients and/or their families had to give up their hobbies because dialysis was too exhausting and time-consuming. One patient stated that the incompatibility of work and dialysis also had an adverse impact on her financial situation, which in turn increased the burden caused by the transport costs.

## 4. Discussion

Haemodialysis is the most common form of dialysis in Switzerland [2] and also worldwide [8]. For patients who require dialysis in a centre, this life-prolonging measure can only be guaranteed if they are able to travel to the centres on a regular basis. Transport to the centre is therefore of major relevance. However, the associated costs can be a particular burden for patients, impacting their socioeconomic situation. In this context, our study gathered first national data on transport costs in Switzerland, combining qualitative and quantitative data. While the quantitative online survey provided a comprehensive database, the qualitative interviews allowed for the inclusion of subjective impressions and personal aspects from patients and their relatives. The involvement of patients, relatives, and healthcare professionals at all stages of the project through the Sounding Board has further enhanced the quality of research and deepened the understanding of the challenges related to transport costs and their impact.

The financial situation of dialysis patients is challenging. Our study found that 31% of respondents have an equivalent income of less than CHF 3000, which is significantly lower than the Swiss average of CHF 4000 reported by the Swiss Federal Statistical Office [26]. Another 39% have an equivalent income between CHF 3000 and 5000. This indicates that many dialysis patients have a comparatively low income. The results of the Canadian Kidney Foundation report (2018) underline the significant impact that dialysis can have on household income: almost 50% of respondents reported a reduction in their annual income, with two-thirds of those affected experiencing a reduction of up to 40%. It is therefore not surprising that 41% of the dialysis patients surveyed were below the Canadian Low Income Cut-Off (LICO), compared to 8 - 14% of the general population [27]. The fact that dialysis is very time-consuming, with four treatments a week [2,5], and that patients often have multiple comorbidities [3], leading to a restricted or absent ability to work [9,10], certainly contributes to this result.

The Canadian report shows that the average out-of-pocket costs associated with dialysis can be a significant proportion of total annual household income [27]. Transport costs are thereby an important factor [27,28]. Although home dialysis could help alleviate this problem, it is not the answer as only a small proportion of patients have access to it today and several barriers need to be overcome, such as inadequate funding, patients’ fear of adverse events or psychosocial problems, or caregivers’ burden of responsibility [5]. Our study revealed that the mean dialysis-related transport costs in Switzerland were CHF 2925 (IQR 1170–6006, EUR 3110, IQR 1244–6385). Although a comparison of the data from this study with those from other countries is severely limited by different factors such as the social and healthcare systems, cost of living, and the calculation of transport costs, certain similarities can be observed. In line with the findings of the Dutch study by Mohnen et al. (2019), our study revealed considerable variability in annual transport costs for dialysis between patients [14]. This variability may be explained by the fact that in our study, we observed variability in the distances travelled, and that costs varied considerably depending on the mode of transport used. The latter result was also found by Allenbach and Pereira (2014) for France, and in both countries, taxi or patient transport services were among the most expensive [15]. However, in contrast to France, our study revealed that patient transport by private car was more expensive in Switzerland.

The online survey showed that the most common mode of transport for dialysis patients was the private car. According to the interviews, this may be explained by the fact that a private car, if available, is significantly cheaper than, for example, volunteer transport services. In addition, survey respondents indicated that they chose the car because of its convenience and shorter travel times (compared to other modes of transport). Another possible explanation may be related to the finding by Lewis et al. (2023), who reported that patients who travelled by car felt more in control of their treatment plans and their lives in general. However, their findings also revealed that independent driving can be stressful for patients due to post-dialysis fatigue or external factors such as challenging weather conditions or long distances [9]. The latter reason, in particular combined with dialysis-related symptoms, was also mentioned by three of the interviewees in our study who had to travel long distances in the mountains and therefore decided to use public transport instead of a private car, despite longer travel times.

As outlined in the background, high transport costs, especially without adequate financial support, can be a significant financial burden that can affect the physical and mental health of the patients [17]. However, this is not an issue limited to dialysis patients. In a systematic review conducted by Larkin et al. (2020), which included 46 qualitative studies and grey literature from six countries, 14 studies found that the high cost of care, mainly related to transport costs and cost of parking at healthcare facilities, had a negative impact on the well-being of persons with multiple chronic conditions. This was reflected in feelings of stress, worry, frustration, and upset, mainly as a result of not being able to afford access to healthcare and related necessities [28].

The fear of not being able to use a private car or public transport due to deteriorating health, and then not being able to afford patient transport, also emerged in our qualitative interviews. Patients wondered what would happen in such situations. This concern is underlined by the extreme example of an interviewee who used to pay CHF 140 per dialysis session for a volunteer transport service, which would end up amounting to CHF 21,840 per year for three sessions per week if he had not been able to travel by train. This example demonstrates that transport is a crucial aspect of dialysis treatment. It also highlights the need to address inequalities in access to dialysis facilities [29].

The results of the online survey showed that one-third of the patients received financial support for travel costs, mainly provided by compulsory health insurance. In Switzerland, compulsory health insurance, with an average monthly premium of CHF 375 in 2023 [30], covers 50% of the costs of medically indicated patient transport if the patient’s state of health does not allow transport by other means, but only up to a maximum of CHF 500 per year [18], i.e., covering a only small fraction. It is therefore not surprising that 50% of our survey respondents were concerned about the incurred transport costs. In addition, almost half of the respondents reported having to financially restrict themselves in their daily lives due to these costs. The interviews illustrated that these restrictions could have a detrimental effect on several aspects of life, such as social participation. This was also shown in studies included in Larkins Review and the Canadian report [27,28]. Participants also experienced shame and stigma due to their financial situation [28]. In contrast, no such experiences were reported in our interviews.

Financial counselling could help dialysis patients counteract negative effects of financial constraints. However, the online survey showed that only half of the respondents had received such support. The majority of those who had not received counselling expressed a desire to do so, highlighting the need for such a service. This desire was also evident in discussions with the Sounding Board. To support patients appropriately and to identify the need for financial support at an early stage, the healthcare professionals of the Sounding Board emphasised the importance of discussing the financial burden of transport with all patients. Ng et al. (2021) also recommend providing appropriate clarifications at the beginning and during the course of dialysis treatment [17]. However, the results of a qualitative study by Patel et al. (2015) found that 75% of the physicians surveyed never or rarely discussed financial burden with their chronically ill patients, and when they did, it was most often initiated by the patients [31]. This suggests that counselling should take place in an additional setting. As confirmed by the Sounding Board, Switzerland currently lacks a specialised contact point for social and financial counselling of dialysis patients. The consequences of this were evident in the interviews with patients and relatives who reported that they were passed from one contact to another without receiving the requested information or being provided with the support that they needed.

In addition to the financial impact, this study also investigated the impact of transport on the social situation of patients. The online survey revealed that approximately one third of the respondents were dependent on private (e.g., friends, family) or professional (e.g., taxi drivers, transport service) assistance for transport. The high mean age of patients and the presence of multiple chronic conditions in a quarter of the Swiss dialysis population, as described by Ambühl (2017), may explain this finding [3]. This conclusion is supported by the fact that physical limitations not related to dialysis were frequently mentioned as a reason for using transport services, both in the online survey and in the interviews.

It should be noted that most survey participants were satisfied with their transport situation. On the one hand, this may be related to the findings of the survey and the interviews that the that transport had either no or a positive impact on their relationships. This was confirmed in the interviews, where patients talked about friendships and partnerships that were strengthened during the travel time. In contrast to the findings of Lewis et al. (2023), the interviewees in our study did not feel that they were a burden on their relatives at the time of the interviews but did express concern that they might become a burden at some point. Similarly, the relatives who participated in our study did not perceive these care tasks as burdensome and appreciated the time they were able to spend with the dialysis patient. Nonetheless, it is important to consider the health and well-being of the carers, as different studies show that caregiving can have both positive (e.g., experience of personal growth) and negative effects (e.g., social isolation, elevated level of burden, stress) [12,13,32]. On the other hand, our participants may be satisfied with their transport situation, as the interviews revealed that they did not experience problems with public transport as described in the study by Lewis et al. (2023). This may be due to the well-developed and reliable public transport system in Switzerland, which serves as a viable alternative to private cars. However, as mentioned above, the patients interviewed expressed concern about having to rely on transport services if they were unable to use public transport or their private car, particularly due to the high costs and lack of flexibility of these services. This again highlights the fact that transport to the dialysis centres is an integral part of the overall dialysis treatment.

### Limitations

First, we aimed to recruit interview partners from different backgrounds, but were unable to include a user of a transport service. However, this perspective was included in the national online survey. Second, although some parameters are consistent with the dialysis registry data, the study population is not fully representative of the Swiss dialysis population. Furthermore, due to the sampling strategy chosen here, there might be a “selection bias”, i.e., persons with a strong interest in the topic may have been more likely to respond. Third, excluding missing data instead of using imputation may also introduce bias into the analysis. Fourth, the questionnaire was only available online, which may have been a barrier for some. However, the high number of participants who completed the survey with assistance indicates that this barrier was overcome.

## 5. Conclusions

Haemodialysis is a life-sustaining treatment requiring regular travel to a dialysis centre, making transport a critical component of ensuring access to care. This study provides the first set of national data on dialysis-related transport costs in Switzerland and highlights the significant challenges faced by patients in this regard. Although most respondents were satisfied with their current transport situation, transport costs impose a significant financial strain on dialysis patients, who often have low incomes and whose health insurances provide only a limited coverage of transport costs. Half of the participants experienced the expenses as burdensome or limiting, forcing patients and their families to make financial sacrifices to afford the transport. Despite an identified demand for financial counselling, there is a lack of such support in Switzerland. Based on these findings, it is recommended to introduce a dedicated counselling service to provide patients with essential information on transport options and financial support options. Future work should explore ways to establish standardised and transparent procedures enabling dialysis patients to access financial support to alleviate transport cost in Switzerland. Moreover, it is recommended to integrate transport needs in the overall planning of dialysis care to ensure comprehensive support for patients.

## Figures and Tables

**Figure A1:**
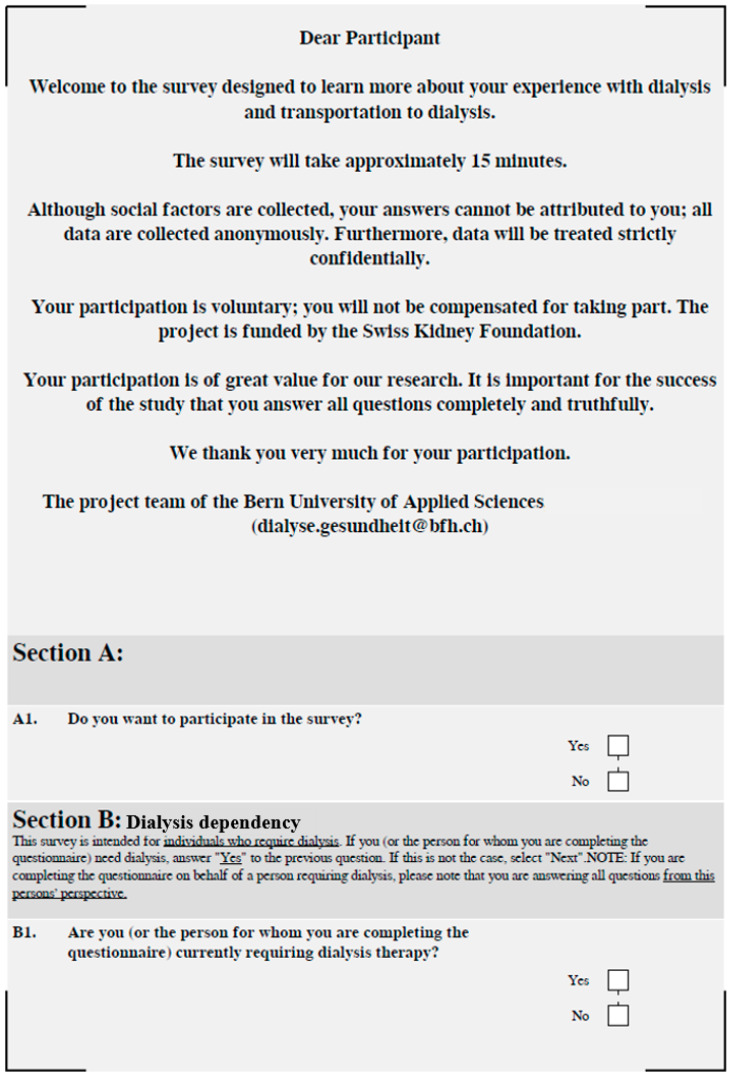

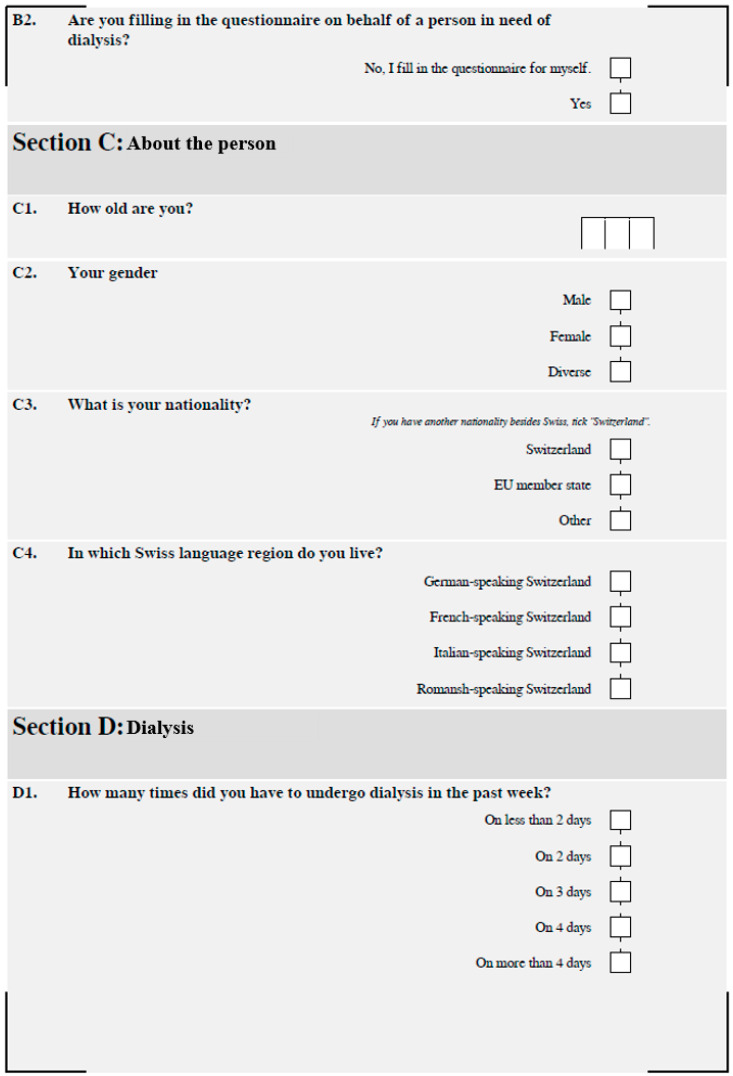

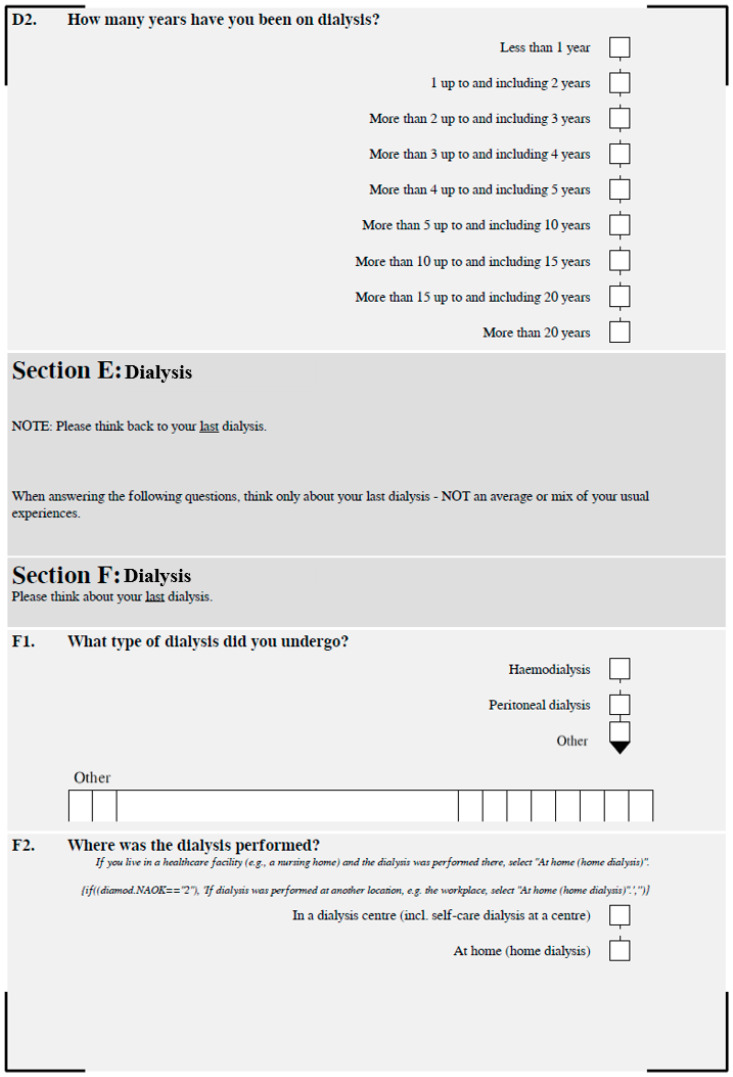

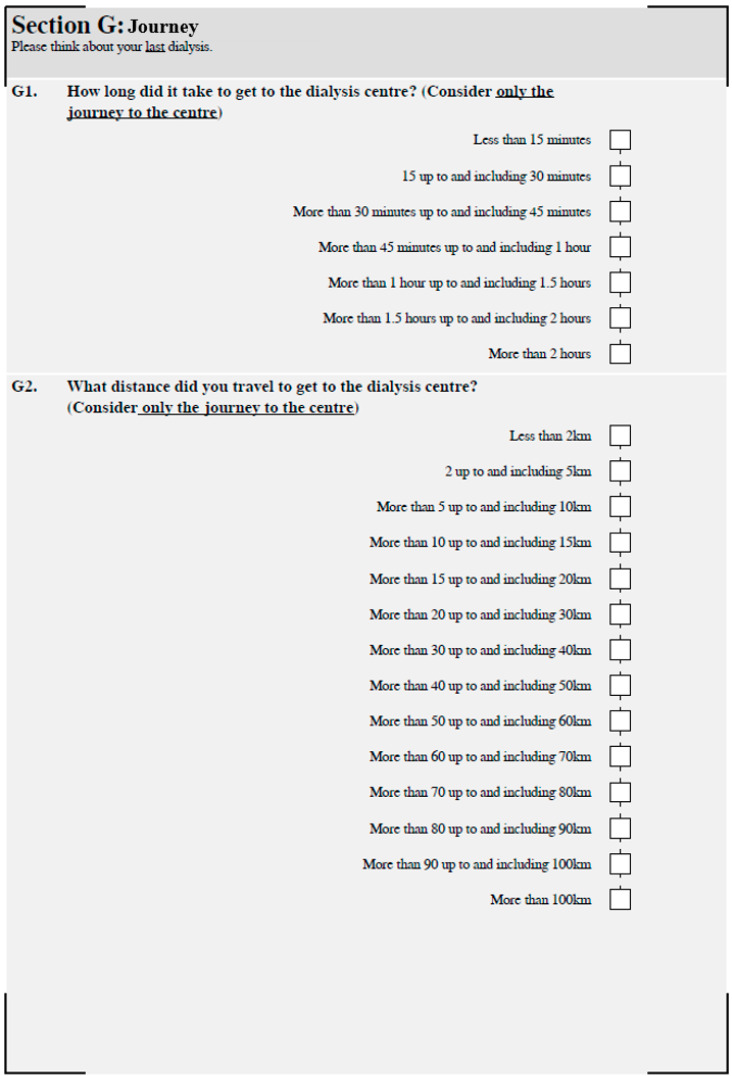

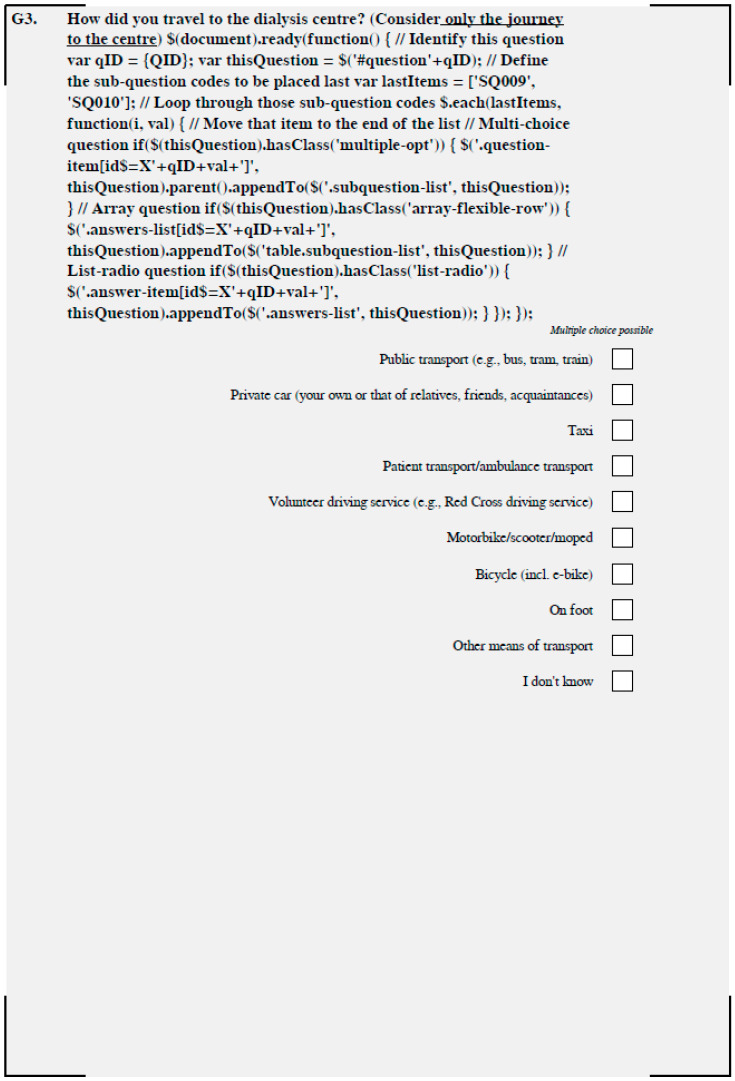

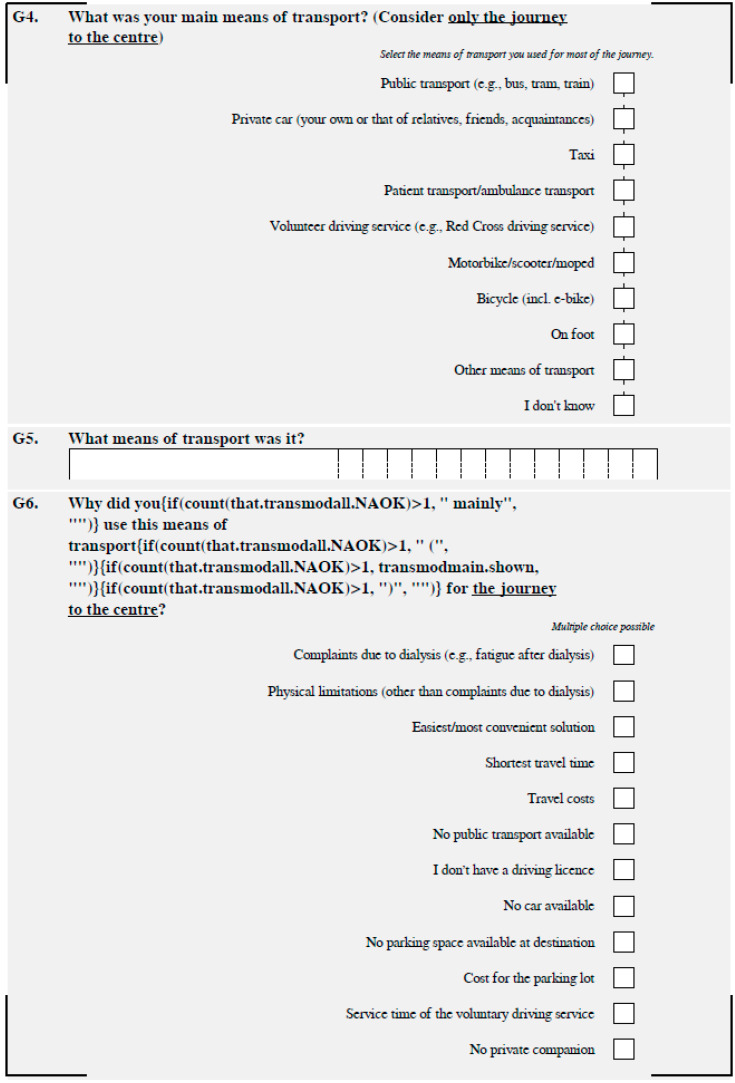

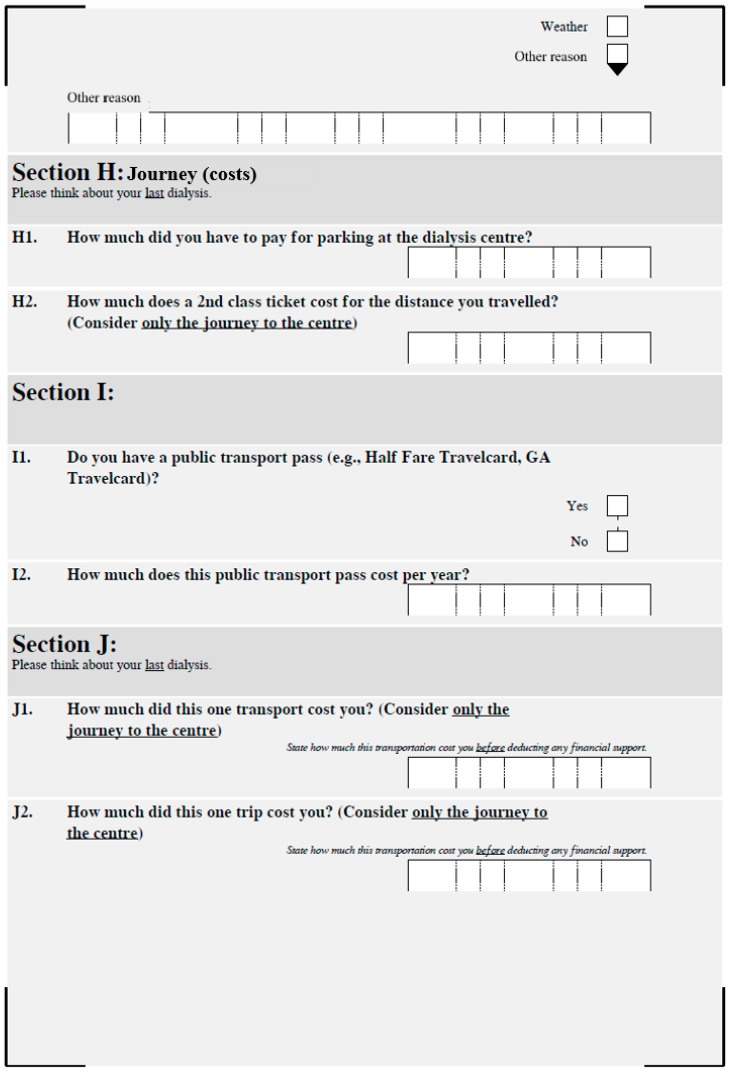

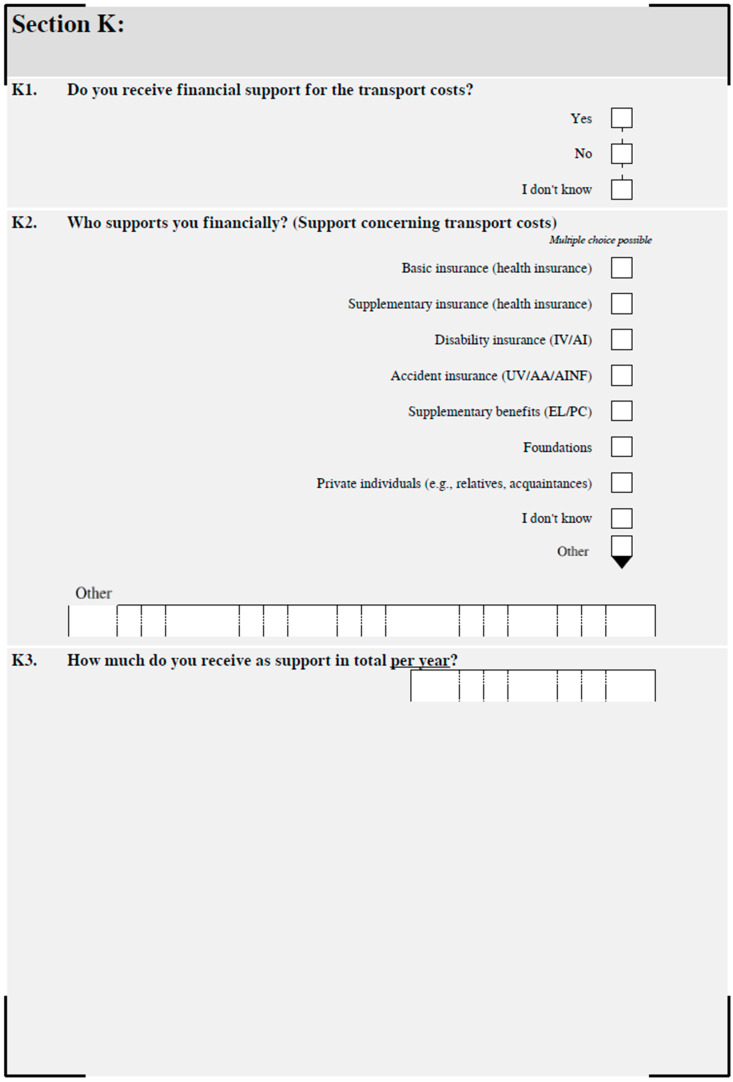

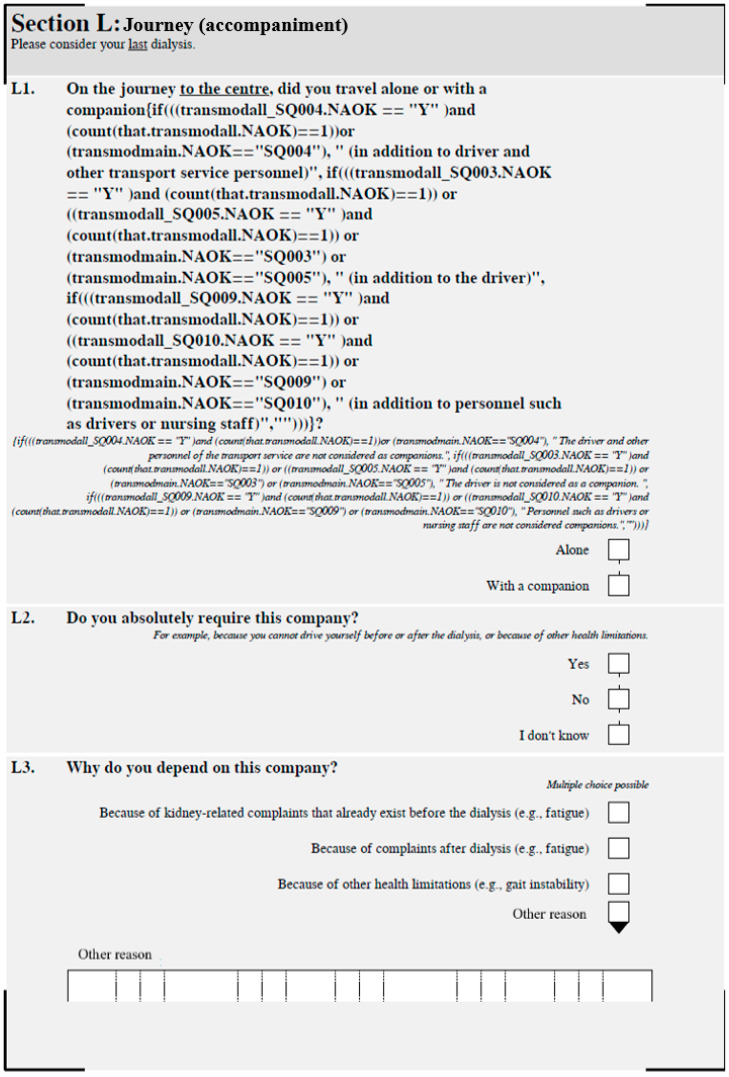

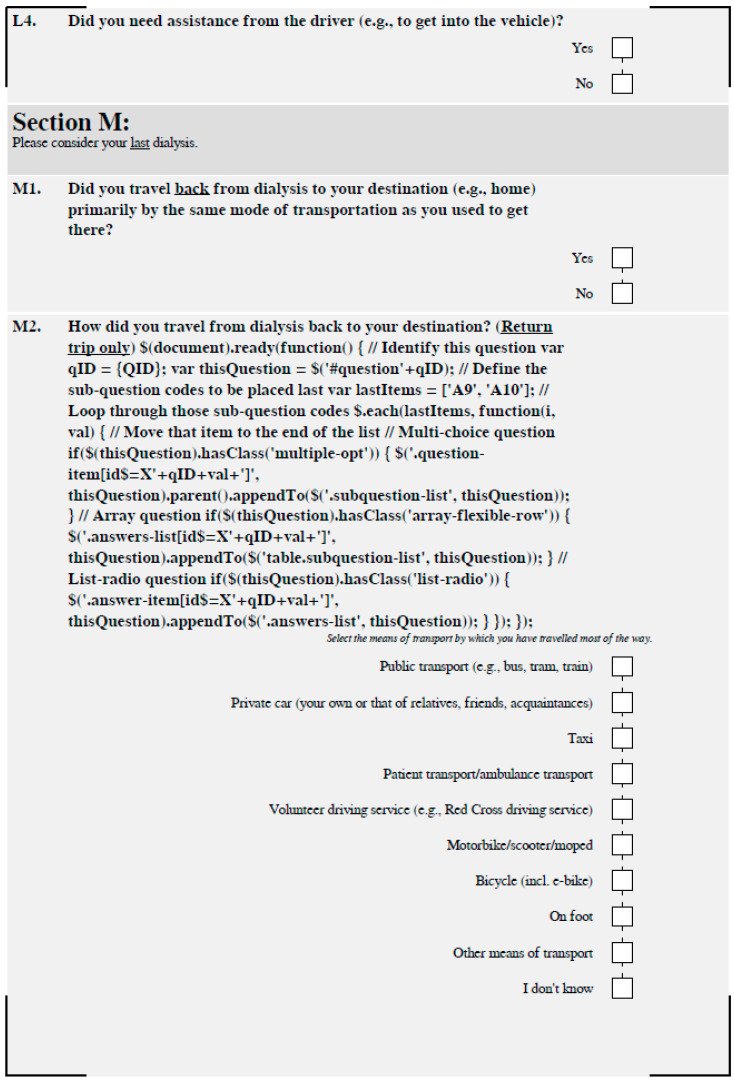

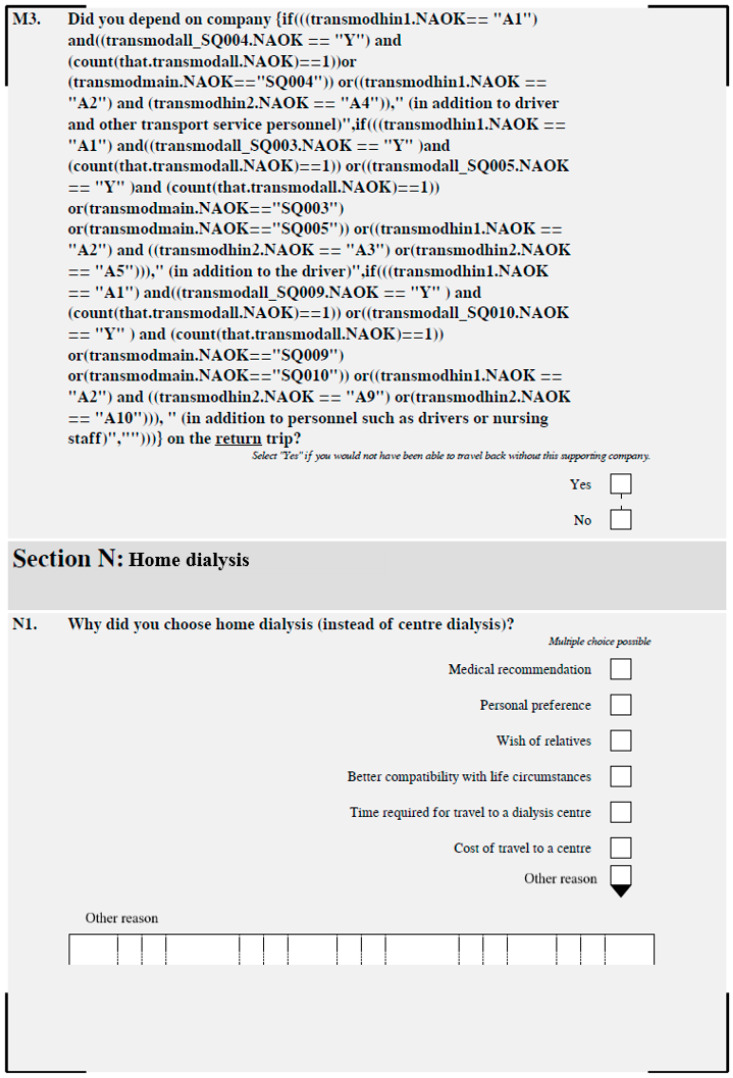

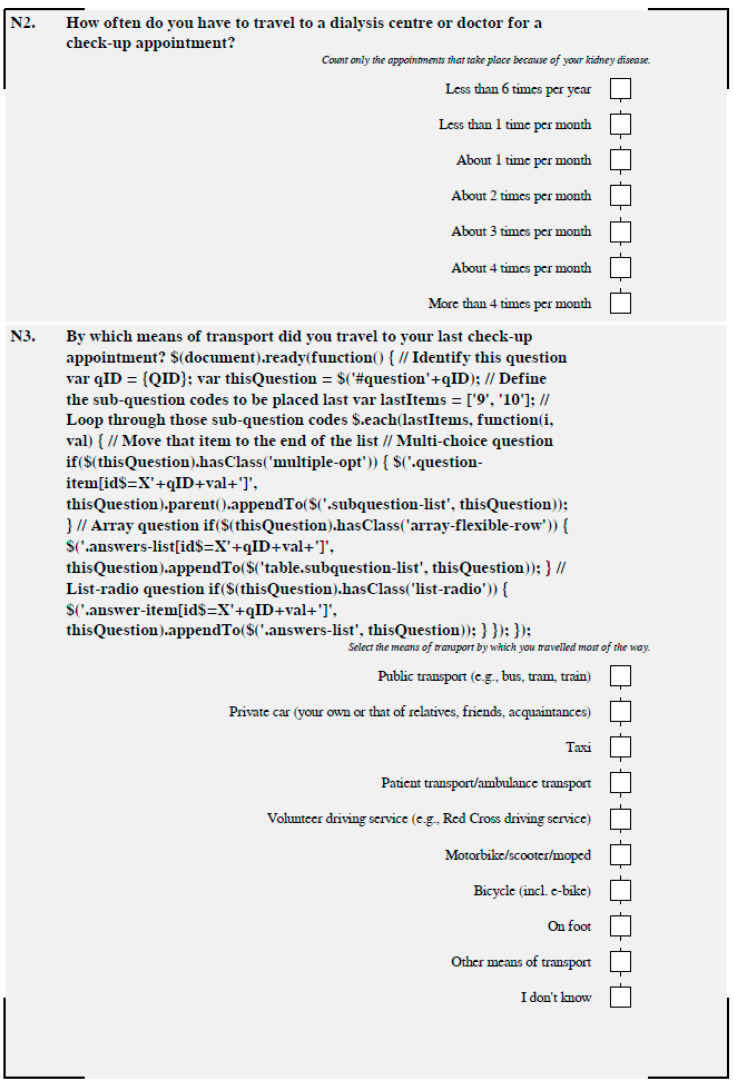

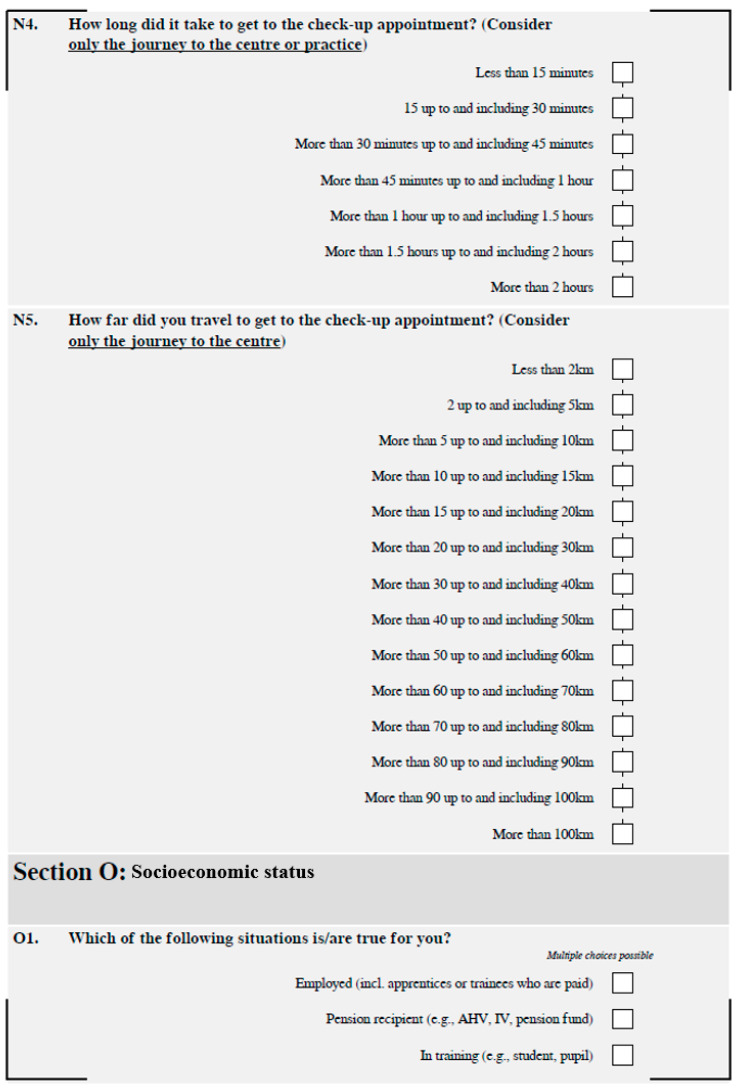

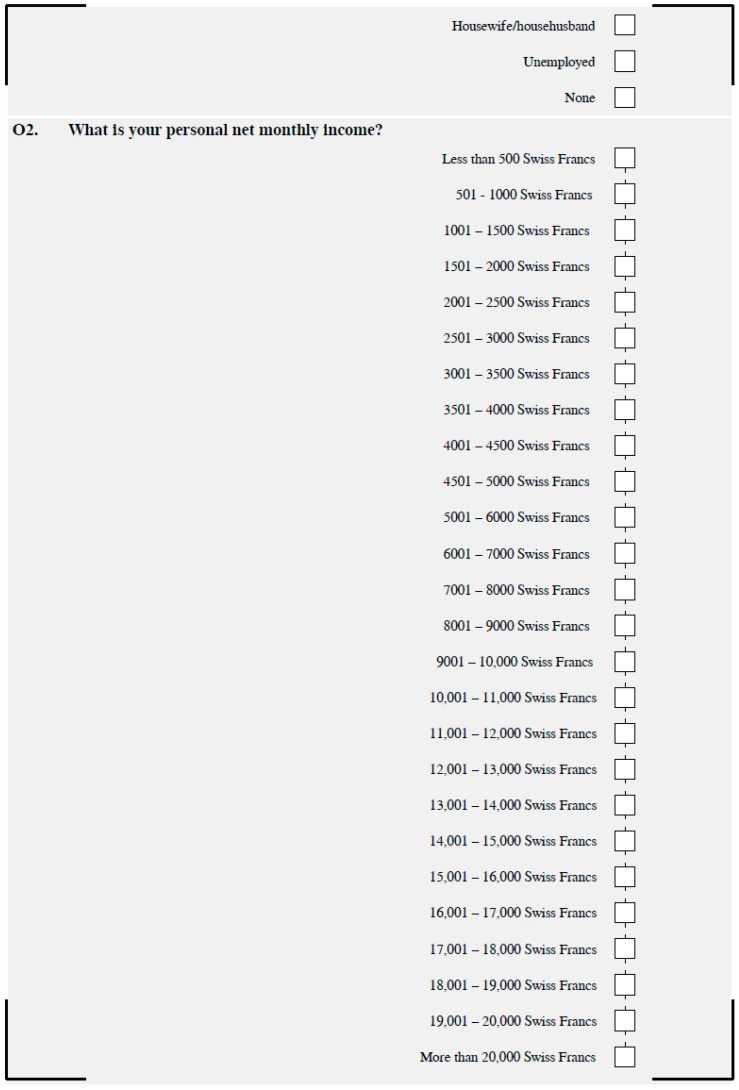

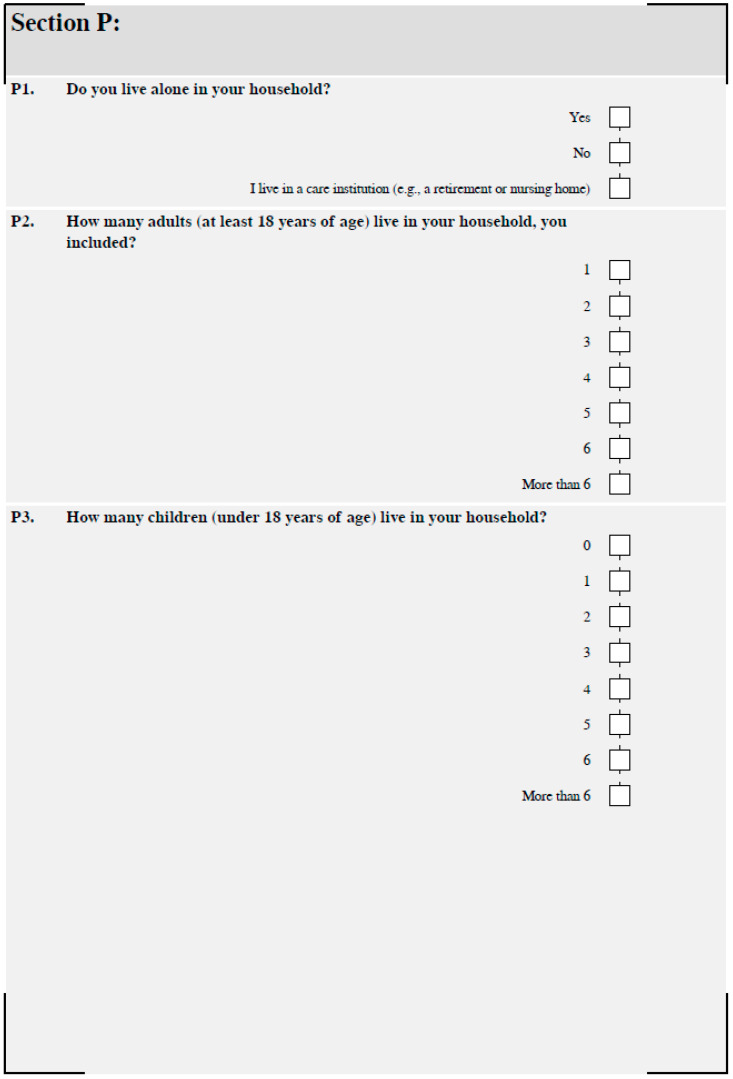

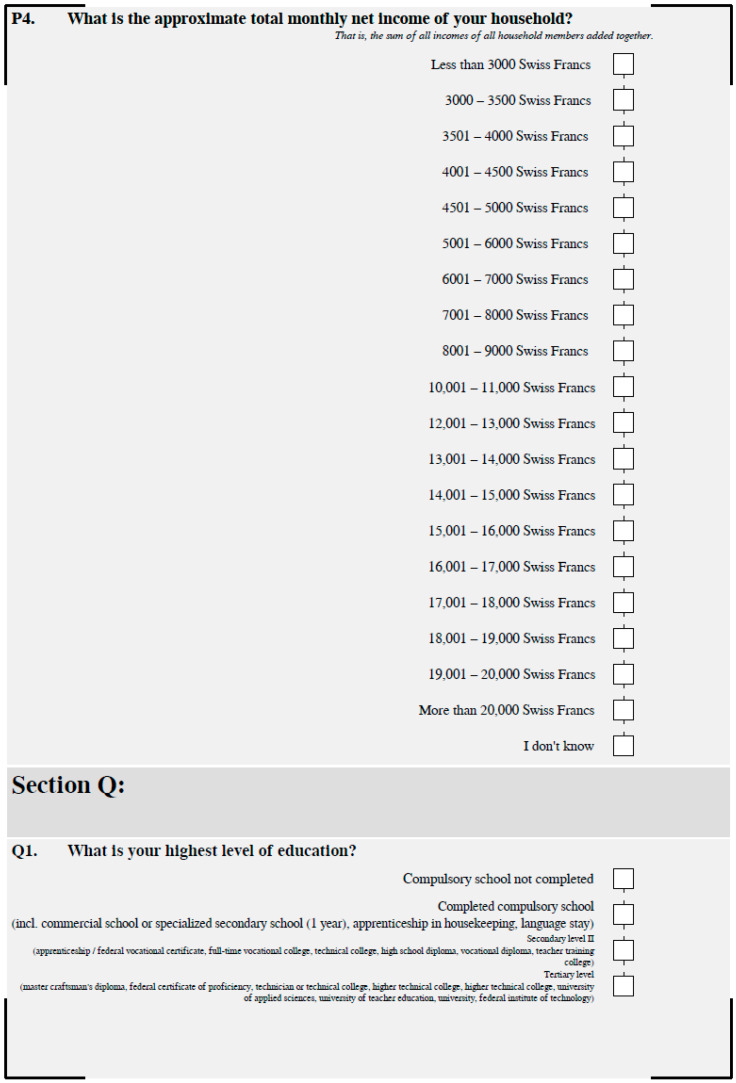

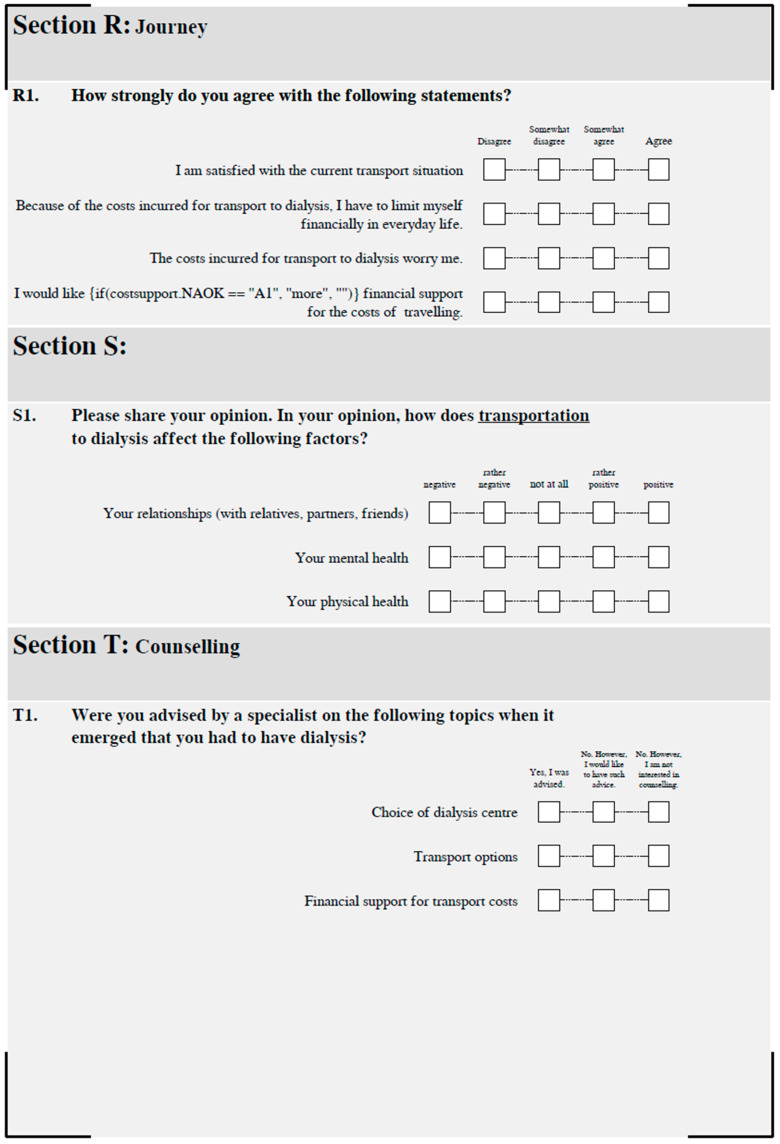

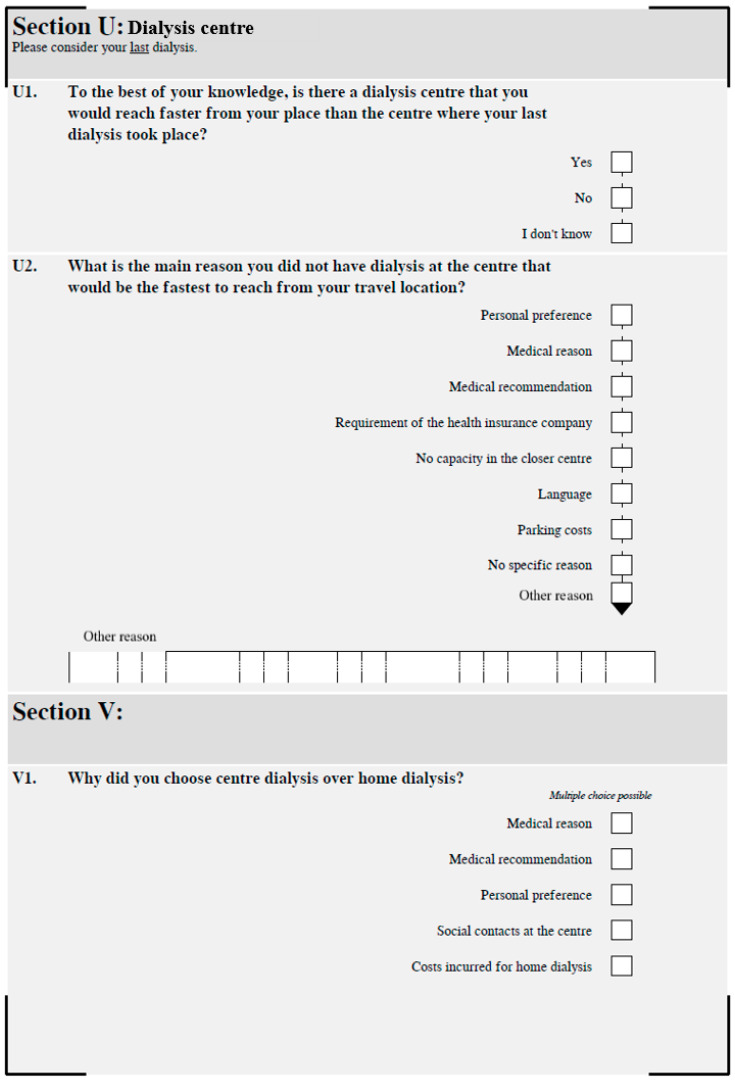

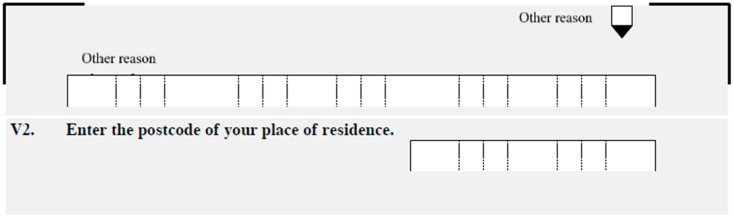

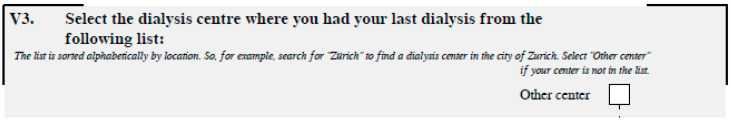

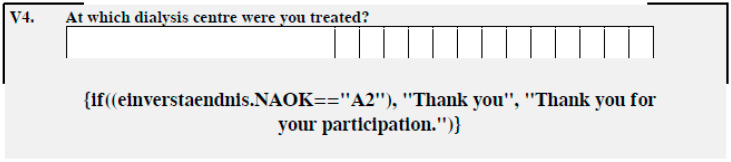
English version of the national online survey on transport costs in Switzerland.

## Data Availability

The data presented in this study are available on reasonable request from the corresponding author due to privacy and ethical restrictions.

## Appendix A

**Table A1:** Categories and subcategories of the qualitative analysis and their impact on the online survey.

Category	Subcategory	Questions in the Online Survey That Were Influenced by the Interview
Journey to the dialysis centre	Choice of means of transport	G6
	Satisfaction with means of transport	R1
	Choice of dialysis centre	U1, U2
	Organisation of the transport	
Influence of transport on the financial situation of dialysis patients	Transport costs	H1
	Financial impact	R1, T1
Effects of haemodialysis on the private life of dialysis patients	Employment	
	Partnership	
	Friendship and family	S1
	Leisure activities	R1
	Household	
	Place of residence	
Influence of transport and dialysis on health	Mental health	S1
	Physical health	L2-4, M3, S1
Influence of transport on close relatives (patient view)	Organising everyday life (household, work)	
	Partnership	S1
	Friendship and family	
Influence of transport on close relatives (relatives view)	Organising everyday life (household, work, holiday)	
	Partnership	
	Friendship and family	
Wishes for the future	Organisation of transport, transport, and transport cost	
	Dialysis centres	
	Medical care and health	

The categories of the qualitative analysis were defined deductively based on the literature and refined inductively on the basis of the content of the interviews. Themes that emerged as central in the analysis of the interviews were incorporated into the formulation of the questions or response options in the online survey.

## Appendix B

**Table A2:** Calculation basis for the different modes of transport.

Mode of Transport	Required Information			Calculation of Costs/Year
	Collected Data	Additional Data ^4^		
	A	B	C	
**Public transport**	Cost of 2nd class ticket		Dialysis treatments/year	2A×C If 2A×C > CHF 3860: Costs = CHF 3860 (Assumption: the full price of the annual travel pass has been paid ^1^)
**Private car**	Distance travelled in kilometres	Costs/vehicle kilometre ^2^	Dialysis treatments/year	A×B×C
**Taxi**	Price for outward journey		Dialysis treatments/year	2A×C
**Patient transport**	Price for outward journey		Dialysis treatments/year	2A×C
**Volunteer transport** **service**	Price for outward journey		Dialysis treatments/year	2A×C
**Motorbike**	Distance travelled in kilometres	Costs/vehicle kilometre ^3^	Dialysis treatments/year	A×B×C
**Bicycle**			Dialysis treatments/year	Assumption: 0
**On foot**				Assumption: 0
**Other**	Price for outward journey			A×2

^1^ Costs for the 2023 annual travel pass valid on the entire Swiss public transport network. See SBB: Alle GA-Preise im Überblick|SBB. ^2^ According to the Swiss Touring Club TCS (2023), a standard car costs CHF 0.75 per vehicle kilometre. ^3^ According to the Swiss Federal Statistical Office BFS (2019), the cost per vehicle kilometre for motorcycles/mopeds is 1.42 times higher than for a car. In 2023, this corresponds to a cost of CHF 1.0625 per vehicle kilometre. ^4^ Questions in which costs were collected as free text answers: “How many francs did this one trip cost you (outward journey only)?”; “How many francs did this one trip cost you (outward journey only)?”; “How many francs does a full 2nd class ticket cost for the distance you travelled (outward journey only)?”.

## Appendix D

**Table A3:** Comparison of the online survey data with the data from the SRRQAP dialysis register.

		Survey					Registry (Year 2021)					Comparison
Variable		N	Mean/Proportion	SD	Min	Max	N	Mean/Proportion	SD	Min	Max	*p*-Value
Age		368	70.1	14.2	21	94	4290	69.7	14.3	2.8	97.8	0.601 1
Gender		369										0.002 2
	Male	250	67.8%				2823	65.8%				
	Female	119	32.2%				1467	34.2%				
Nationality		370										<0.001 3
	Swiss	312	84.3%				3204	74.7%				
	EU citizen	40	10.8%				585	13.6%				
	Other	18	4.9%				501	11.7%				
Swiss language region		366										<0.001 3
	German	234	234	63.9%			2870	66.9%				
	French	27	27	7.4%			1114	26%				
	Italian	105	105	28.6%			306	7.1%				
Years requiring dialysis		368	4.3	4.2	0.5	20	4290	4.2	4.2	0.0	44.8	0.613 1
Dialysis per week		370	2.9	0.3	1.0	5.0	4290	3.0	0.2	1.0	6.0	0.017 1

Survey N total = 371; ^1^ Z-test; ^2^ Z-test for proportions; ^3^ Chi-Square Goodness-of-Fit test; the variables “years requiring dialysis” and “dialyses per week”, initially assessed categorically in the survey, were converted to continuous variables based on assumptions for comparison with registry data. SRRQAP = Swiss renal registry and quality assessment program.

## Appendix E

**Table A4:** Sample characteristics of the interviewed patients.

Patient	Gender	Age	Years of Dialysis	Location of the Interviews	Residential Area
Patient 1	female	74	2	hospital	rural
Patient 2	male	82	4	hospital	urban
Patient 3	male	82	2.5	patient’s home	urban
Patient 4	male	83	0.5	hospital	rural
Patient 5	female	50	10	hospital	rural
Patient 6	male	82	0.5	hospital	rural

## Appendix F

**Table A5:** Annual transport costs by mode of transport.

Mode of Transport	N	Mean	SD	Pctl (25)	Median	Pctl (75)
Public transport	40	1957	1182	874	1560	2737
Private car	179	3011	2576	819	1755	4095
Taxi	12	10,461	7336	6552	9828	14,040
Patient transport	54	10,200	11,836	2496	6396	12,480
Volunteer transport service	47	9381	5585	874	7488	12,480
Motorcycle	3	1879	2005	746	1160	2652
Bicycle	3	0	0	0	0	0
On foot	7	0	0	0	0	0
Other	3	4680	8106	0	0	7020

Table shows transport costs by mode of transport. Cost of “bicycle”/“on foot” = 0 by definition. SD = standard deviation; Pctl = percentile; N total = 371.

## Appendix G

**Table A6:** Transport costs by various groups.

	Annual Transport Costs (CHF)						
Variable	N	Mean	SD	Pctl (25)	Median	Pctl (75)	*p*-Value
**Age**	347						0.089 ^1^
20–44	21	3783	6425	811	1310	2925	
45–64	87	5268	7616	1755	2925	5850	
65–74	86	4561	5604	833	2340	6142	
75+	153	5381	6453	1170	2995	7176	
**Gender**	346						0.034 ^2^
Male	233	4763	6780	874	2652	5850	
Female	113	5592	6104	1560	4095	7488	
**Financial support**	335						<0.001 ^2^
Yes	92	8295	9402	2730	5850	9360	
No	243	3657	4452	819	1755	4680	
**Years requiring dialysis**	289						0.407 ^1^
≤5	199	4747	6020	1033	2925	5850	
>5–10	58	5069	4978	1755	3998	6679	
>10–15	18	8054	14,060	1804	4232	7176	
>15–20	8	3890	3873	1482	2925	4241	
>20	6	4293	3487	1310	4048	6376	
**Language region**	343						<0.001 ^1^
German	220	5607	6254	1755	3860	7176	
French	27	3622	3067	1346	2925	4649	
Italian	96	4011	7659	819	1560	4446	
**“Was advised on the choice of centre”**	344						0.029 ^2^
Yes	257	5556	7181	1310	2925	7176	
No	87	3477	3738	1079	2184	4388	
**“Was advised on transport options”**	346						0.007 ^2^
Yes	184	6186	7957	1381	3900	7898	
No	162	3792	4161	1163	2215	5850	
**Living situation**	348						0.058 ^1^
Alone	129	4867	5869	1248	2925	5850	
Not alone	198	4286	4602	1039	2925	5850	
Care institution	21	13,036	15,599	1560	9360	15,600	
**Monthly equivalent income (net CHF)** ^3^	322						0.564 ^1^
≤3000	100	5222	6143	819	2652	7696	
3001–5000	126	5378	7562	1443	2960	6240	
5001–10,000	80	4748	5075	1706	2925	5948	
>10,000	16	2964	2581	1755	1852	3114	
**Individual monthly income (net CHF)**	316						0.846 ^1^
≤3000	185	5069	5380	874	3744	7176	
3001–5000	85	5598	8469	1248	2925	6240	
5001–10,000	40	3986	4840	1560	2067	5850	
>10,000	6	4069	3629	1755	2340	4709	
**Main mode of transport**	348						<0.001 ^1^
Public transport	40	1957	1182	874	1560	2738	
Private car	179	3011	2576	819	1755	4095	
Taxi	12	10,460	7336	6552	9828	14,040	
Patient transport	54	10,200	11,836	2496	6396	12,480	
Volunteer transport service	47	9381	5585	5460	7488	12,480	
Motorcycle	3	1879	2005	746	1160	2652	
Bicycle	3	0	0	0	0	0	
On foot	7	0	0	0	0	0	
Other	3	4680	8106	0	0	7020	
**“I am satisfied with the current transport situation”**	343						0.202 ^1^
Disagree	32	6754	7688	1606	4388	8326	
Somewhat disagree	56	5264	6013	1755	2925	5850	
Somewhat agree	81	4628	4604	936	2995	6240	
Agree	174	4797	7242	1072	2574	5850	
**“Because of the costs incurred for transport to the dialysis centre, I have to limit myself financially in everyday life”**	339						<0.001 ^1^
Disagree	141	3702	5450	819	1755	4095	
Somewhat disagree	50	3890	2733	1755	2960	5850	
Somewhat agree	87	6555	8821	1755	4095	7995	
Agree	61	6961	6695	1755	5616	8736	
**“I am worried about the cost of transport to dialysis”**	340						<0.001 ^1^
Disagree	107	4283	8221	819	1755	3802	
Somewhat disagree	61	4272	5989	1755	2925	5850	
Somewhat agree	88	4828	4675	1737	3568	6240	
Agree	84	6964	6094	2496	5850	8892	
**“I wish for [more] financial support for the incurred transport costs”**	340						<0.001 ^1^
Disagree	59	4352	7211	819	1755	4232	
Somewhat disagree	17	3217	3829	819	1755	2925	
Somewhat agree	60	3506	3390	1404	2067	4241	
Agree	204	5915	7157	1755	3998	7800	

^1^ Kruskal–Wallis rank sum test. ^2^ Mann–Whitney U test. ^3^ To calculate the monthly equivalent income, the household income, the number of adults, and the number of children in the household were asked in the online questionnaire. It was calculated as follows: Equivalent income = household income/((1 × number of adults) + (0.5 × children)), assumption: all children ≥ 15 years old. The table shows how the costs vary across different characteristics and other factors. CHF = Swiss Francs; SD = standard deviation; Pctl = percentile; N total = 371.

## References

[1] Kovesdy C.P. (2022). Epidemiology of chronic kidney disease: An update 2022. Kidney Int. Suppl..

[2] Swiss Renal Registry and Quality Assessment Program SRRQAP SRRQAP. https://www.swissnephrology.ch/srrqap/.

[3] Ambühl P.M. (2017). Aktuelle Erkenntnisse zur Schweizer Dialysepopulation. Hausarzt Prax..

[4] Charles C., Ferris A.H. (2020). Chronic Kidney Disease. Prim. Care.

[5] Torreggiani M., Piccoli G.B., Moio M.R., Conte F., Magagnoli L., Ciceri P., Cozzolino M. (2023). Choice of the Dialysis Modality: Practical Considerations. J. Clin. Med..

[6] Chan C.T., Blankestijn P.J., Dember L.M., Gallieni M., Harris D.C.H., Lok C.E., Mehrotra R., Stevens P.E., Wang A.Y.-M., Cheung M. (2019). Dialysis initiation, modality choice, access, and prescription: Conclusions from a Kidney Disease: Improving Global Outcomes (KDIGO) Controversies Conference. Kidney Int..

[7] Bello A.K., Okpechi I.G., Levin A., Ye F., Saad S., Zaidi D., Houston G. (2023). ISN–Global Kidney Health Atlas: A Report by the International Society of Nephrology: An Assessment of Global Kidney Health Care Status Focussing on Capacity, Availability, Accessibility, Affordability and Outcomes of Kidney Disease. https://www.theisn.org/wp-content/uploads/media/ISN%20Atlas_2023%20Digital_REV_2023_10_03.pdf.

[8] United States Renal Data System (2018). 2018 USRDS Annual Data Report: Epidemiology of Kidney Disease in the United States. Bethesda, MD. https://www.niddk.nih.gov/-/media/Files/USRDS/2018-Previous-ADR/Volume-2/Slides/v2_c11_intcomp_18_slides.pptx.

[9] Lewis R.A., Bohm C., Fraser F., Fraser R., Woytkiw L., Jurgutis S., Rubin M., Smith G., Buenafe J., Verdin N. (2023). Transportation Burden Associated with Hemodialysis in Canada: A Qualitative Study of Stakeholders. Kidney Med..

[10] AlHejaili F., Hashmi M.N., Alsuwaida A., Ankawi G.A., ALMehaideb S.A., Alsuwaida A.A., AlZahrani M.T., Shehadah A.E., AlNasser H.A. (2024). Burden of Chronic Hemodialysis on the Ability to Work: Time for Action. Avicenna J. Med..

[11] McLean R.M., Xie Z., Nelson V., Nosa V., Thein H., Po’e-Tofaeono A., Walker R., Wyeth E.H. (2021). Experiences of New Zealand Haemodialysis Patients in Relation to Food and Nutrition Management: A Qualitative Study. Nutrients.

[12] Beanlands H., Horsburgh M.E., Fox S., Howe A., Locking-Cusolito H., Pare K., Thrasher C. (2005). Caregiving by family and friends of adults receiving dialysis. Nephrol. Nurs. J..

[13] Alshammari B., Noble H., McAneney H., Alshammari F., O’Halloran P. (2021). Factors Associated with Burden in Caregivers of Patients with End-Stage Kidney Disease (A Systematic Review). Healthcare.

[14] Mohnen S.M., Van Oosten M.J.M., Los J., Leegte M.J.H., Jager K.J., Hemmelder M.H., Logtenberg S.J.J., Stel V.S., Hakkaart-van Roijen L., De Wit G.A. (2019). Healthcare costs of patients on different renal replacement modalities—Analysis of Dutch health insurance claims data. PLoS ONE.

[15] Allenbach D., Pereira O. (2015). Analyse de la demande de transport des patients dialysés en Lorraine. Santé Publique.

[16] Ferguson T.W., Harper G.D., Milad J.E., Komenda P.V.J. (2022). Cost of the quanta SC+ hemodialysis system for self-care in the United Kingdom. Hemodial. Int..

[17] Ng M.S.N., Chan D.N.S., Cheng Q., Miaskowski C., So W.K.W. (2021). Association between Financial Hardship and Symptom Burden in Patients Receiving Maintenance Dialysis: A Systematic Review. Int. J. Environ. Res. Public Health.

[18] Federal Office of Public Health FOPH Transport-und Rettungskosten. https://www.bag.admin.ch/bag/de/home/versicherungen/krankenversicherung/krankenversicherung-leistungen-tarife/Leistungen-an-Transport-und-Rettungskosten.html.

[19] Krebsliga Schweiz (2019). Transportkoten zu Therapien und Untersuchungen, Bern. https://shop.krebsliga.ch/files/kls/webshop/PDFs/deutsch/transportkosten-zu-therapien-und-untersuchungen-011820902141.pdf.

[20] Jilani H., Rathjen K.I., Schilling I., Herbon C., Scharpenberg M., Brannath W., Gerhardus A. (2020). Handreichung zur Patient*Innenbeteiligung an Klinischer Forschung.

[21] National Kidney Care Audit Patient Transport Survey 2010. https://files.digital.nhs.uk/publicationimport/pub02xxx/pub02700/nati-kidn-care-2010-pati-tran-surv-upda-rep.pdf.

[22] Wong C.K.H., Chen J., Fung S.K.S., Mok M.M.Y., Cheng Y.L., Kong I., Lo W.K., Lui S.L., Chan T.M., Lam C.L.K. (2019). Direct and indirect costs of end-stage renal disease patients in the first and second years after initiation of nocturnal home haemodialysis, hospital haemodialysis and peritoneal dialysis. Nephrol. Dial. Transpl..

[23] Touring Club Switzerland Kilometerkosten—Was kostet mein Auto?. https://www.tcs.ch/de/testberichte-ratgeber/ratgeber/kontrollen-unterhalt/kilometerkosten.php.

[24] Federal Statistical Office FSO (2019). Berechnung der Verkehrsmittelkosten des motorisierten Strassenverkehrs. https://dam-api.bfs.admin.ch/hub/api/dam/assets/7986807/master.

[25] Federal Statistical Office FSO Die häufigsten Hauptsprachen der ständigen Wohnbevölkerung. https://www.bfs.admin.ch/bfs/de/home/statistiken/bevoelkerung/sprachen-religionen/sprachen.assetdetail.24205425.html.

[26] Federal Statistical Office FSO Entwicklung der Einkommensverteilung und -ungleichheit im Jahr 2020. https://www.bfs.admin.ch/news/de/2023-0487.

[27] The Kidney Foundation of Canada (2018). The Burden of Out-of-Pocket Costs for Canadians with Kidney Failure: 2018 Report. https://kidney.ca/KFOC/media/images/PDFs/3-2-1-NAT-Burden_of_Out-of-Pocket_Costs.pdf.

[28] Larkin J., Foley L., Smith S.M., Harrington P., Clyne B. (2021). The experience of financial burden for people with multimorbidity: A systematic review of qualitative research. Health Expect..

[29] Tian F.F., Hall Y.N., Griffin S., Kranze T., Marcella D., Watnick S., O’Hare A.M. (2023). The Complex Patchwork of Transportation for In-Center Hemodialysis. J. Am. Soc. Nephrol..

[30] Federal Department of Home Affairs DHA, Federal Office of Public Health FOPH (2024). Anhaltendes Kostenwachstum führt zu erneuter Prämienerhöhung im Jahr 2025.

[31] Patel M.R., Shah K.S., Shallcross M.L. (2015). A qualitative study of physician perspectives of cost-related communication and patients’ financial burden with managing chronic disease. BMC Health Serv. Res..

[32] Van Hoang L., Green T., Bonner A. (2018). Informal caregivers’ experiences of caring for people receiving dialysis: A mixed-methods systematic review. J. Ren. Care.

